# Lipophilic Shellfish Poisoning Toxins in Marine Invertebrates from the Galician Coast

**DOI:** 10.3390/toxins15110631

**Published:** 2023-10-27

**Authors:** Araceli E. Rossignoli, Begoña Ben-Gigirey, Mónica Cid, Carmen Mariño, Helena Martín, Soledad Garrido, Francisco Rodríguez, Juan Blanco

**Affiliations:** 1 Centro de Investigacións Mariñas (CIMA), Xunta de Galicia, Pedras de Corón s/n, 36620 Vilanova de Arousa, Spainjuan.carlos.blanco.perez@xunta.gal (J.B.); 2 Xefatura Territorial de Vigo, Consellería do Mar, Xunta de Galicia, Concepción Areal nº8, 4, 36201 Vigo, Spain; 3 European Union Reference Laboratory for Monitoring of Marine Biotoxins, Citexvi, Fonte das Abelleiras 4, 36310 Vigo, Spain; bben@aesan.gob.es (B.B.-G.); mcidm@aesan.gob.es (M.C.); frodriguezh@aesan.gob.es (F.R.); 4 Centro Nacional Instituto Español de Oceanografía (IEO-CSIC), Centro Oceanográfico de Vigo, Subida a Radio Faro 50, 36390 Vigo, Spain; garridosoledad21@gmail.com (S.G.)

**Keywords:** emerging, invertebrates, lipophilic toxins, new vectors, NW Spain, sentinels

## Abstract

For the purpose of assessing human health exposure, it is necessary to characterize the toxins present in a given area and their potential impact on commercial species. The goal of this research study was: (1) to screen the prevalence and concentrations of lipophilic toxins in nine groups of marine invertebrates in the northwest Iberian Peninsula; (2) to evaluate the validity of wild mussels (*Mytilus galloprovincialis*) as sentinel organisms for the toxicity in non-bivalve invertebrates from the same area. The screening of multiple lipophilic toxins in 1150 samples has allowed reporting for the first time the presence of 13-desmethyl spirolide C, pinnatoxin G, okadaic acid, and dinophysistoxins 2 in a variety of non-traditional vectors. In general, these two emerging toxins showed the highest prevalence (12.5–75%) in most of the groups studied. Maximum levels for 13-desmethyl spirolide C and pinnatoxin G were found in the bivalves *Magallana gigas* (21 µg kg^−1^) and *Tellina donacina* (63 µg kg^−1^), respectively. However, mean concentrations for the bivalve group were shallow (2–6 µg kg^−1^). Okadaic acid and dinophysistoxin 2 with lower prevalence (1.6–44.4%) showed, on the contrary, very high concentration values in specific species of crustaceans and polychaetes (334 and 235 µg kg^−−1^, respectively), to which special attention should be paid. Statistical data analyses showed that mussels could be considered good biological indicators for the toxicities of certain groups in a particular area, with correlations between 0.710 (for echinoderms) and 0.838 (for crustaceans). Polychaetes could be an exception, but further extensive surveys would be needed to draw definitive conclusions.

## 1. Introduction

Lipophilic toxins (LTs) regulated in the European Union (EU) comprise three groups of polyether compounds [1]. Okadaic acid (OA)-group toxins, which include OA and dinophysistoxins (DTXs) (mainly DTX1, DTX2, and DTX3), are responsible for Diarrhetic Shellfish Poisoning (DSP), a syndrome characterized by abdominal pain, diarrhea, nausea, and vomiting [2]. Nonetheless, alterations in DNA and cellular components, effects on immune and nervous systems, embryonic development, and the potential role of OA as a carcinogenic agent have also been reported [3]. Azaspiracid (AZA)-group toxins comprise several analogs, among which only AZA1, AZA2, and AZA3 are regulated in the EU. AZAs produce neurological symptoms similar to DSP [4,5]. The third group, Yessotoxins (YTXs), are disulfated polyethers with an action mechanism in which second messengers and main intracellular organelles are involved. However, human intoxications related to YTXs have never been reported [6].

Among the LTs, the cyclic imines (CIs) are emerging marine biotoxins characterized by having an imino group as common pharmacophore. They provoke a fast-acting toxicity in mice after intraperitoneal administration [7], resulting in neurotoxic effects. Although CIs occur in microalgae and shellfish worldwide, no human intoxications have been related to their presence in seafood and, so far, they are not regulated in the EU [2,8]. However, the European Food Safety Authority (EFSA), in 2010, requested more data to perform a conclusive risk assessment for consumers [8] and since then more studies on their occurrence in European shellfish have been conducted [9,10,11,12,13].

CIs are divided into several subgroups according to their structure: i.e., pinnatoxins (PnTXs), spirolides (SPXs), and gymnodimines (GYMs), among others. PnTXs are emerging LTs with relative water solubility [14] produced by the dinoflagellate *Vulcanodinium rugosum* [15]. They have been reported in several countries, including some European ones, such as France, Spain, Italy, Portugal, Slovenia, and Norway [10,14,16,17]. PnTXs produce acute neurotoxic effects in mice after oral administration. SPXs comprise more than twenty different analogs, which are produced by the dinoflagellate *Alexandrium ostenfeldii* [18,19] or *A. peruvianum* (synonym of *A. ostenfeldii*) [20]. SPXs were first detected in 1995, in shellfish from Nova Scotia (Canada) [21] and thereafter worldwide in shellfish and phytoplankton. In European coasts, several studies informed later on about their presence [7,11,16,20,22,23]. GYMss are the smallest CIs and only the chemical structures of GYMA, GYMB, GYMC, GYMD [9], GYME, and 16-desmethyl GYMD have been elucidated to date [24]. GYMs were first reported in shellfish in New Zealand [25,26] and later in Tunisia [27,28], South Africa [29], and China [30]. In Europe, there exist several papers documenting the detection of GYMs in shellfish from Italy [7], France [31], Croatia [11,32], and Spain [33].

In Galicia, the presence of non-regulated LTs, such as CIs, has been previously reported by several authors. SPXs were first detected in mussels (*Mytilus galloprovincialis*) and razor clams (*Ensis arcuatus*) in 2005 [22]. The first detection of PnTXs and GYMs in shellfish from Galicia and Cantabria (NorthWest (NW) and North (N) Spain) was in 2019 [17,34], 2021 [33], and 2023 [35], respectively.

It is known that the uptake of algal toxins by filter-feeding shellfish poses a risk to public health; however, non-traditional vectors can accumulate marine biotoxins, also at hazardous levels [36]. There exist very few reports about the presence of regulated marine LTs in non-traditional vectors [37,38,39,40,41,42,43,44,45,46,47]. SPXs, PnTXs, and GYMs have been reported in different shellfish species worldwide [9]. However, very little information is available about their presence in non-traditional vectors. GYMs have been reported in abalones from New Zealand [45], limpets from Lebanon [46], and several invertebrates classes such as gastropods, echinoderms, crustaceans, and cnidaria from NW Spain [35]. SPXs have been found to accumulate in paddle crabs in laboratory assays (*Ovalipes catharus*) [36] and at very low levels in other invertebrates in Portugal [47].

Certain crustaceans, gastropods, cephalopods, echinoderms, and tunicates constitute commercially valuable resources in several EU countries. Therefore, more data on the presence of regulated and emerging LTs in EU non-traditional invertebrate vectors are crucial to performing risk-assessment evaluation studies. These can also provide information on potential accumulation and transfer in the food web, allowing appropriate further monitoring if needed [48,49]. The main aim of this study was to evaluate the presence of regulated and emerging (CIs) toxins in non-traditional invertebrate vectors from NW Spain. Additionally, the possibility of using wild mussels (*M. galloprovincialis*) as sentinel organisms for toxicity in other invertebrates from the same area has been explored.

## 2. Results

### 2.1. LTs in Different Marine Invertebrates Groups

The total number of specimens analyzed for LTs between April 2021 and December 2022 was 1150 (745 bivalves, 191 gastropods, 83 crustaceans, 57 cnidarians, 51 echinoderms, 9 polychaetes, 8 cephalopods, 4 sea squirts, and 2 poriferous, Table S1).

**Figure 1:**
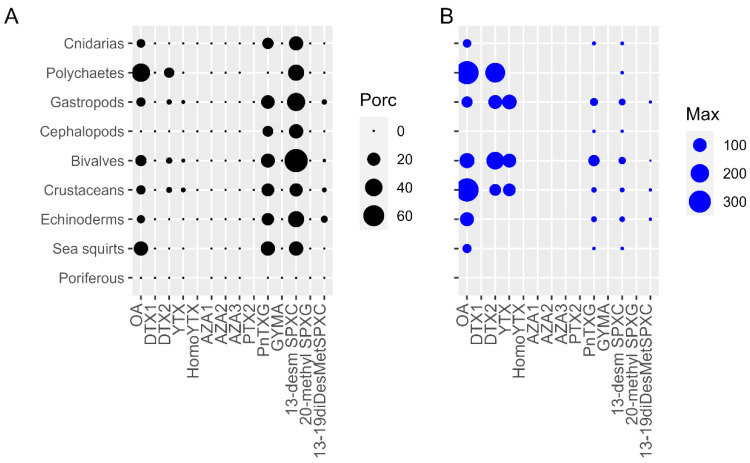
(**A**) Prevalence (%) and (**B**) Maximum concentrations (µg kg^−1^) of the LTs in each group of invertebrates.

The highest prevalence (percentages of samples > limit of detection, LOD) of toxins was found in 13-desmethyl spirolide C (13-desm SPXC) (20.5–75%) and Pinnatoxin G (PnTXG) (12.5–25%) for all groups studied except for SPXs in poriferous and PnTXs in polychaetes and poriferous (Figure 1A). The following most important toxins in prevalence were OA and to a lesser extent DTX2 in polychaetes (44.4% for OA, 11.2% for DTX2), sea squirts (25% for OA), bivalves (13.8% for OA and 2.8% for DTX2), crustaceans (8.4% for OA and 2.4% for DTX2), gastropods (8.4% for OA and 1.6% for DTX2), and echinoderms (5.9% for OA) (Figure 1A). Minor percentages, below 3.9% of 13-19 didesmethyl spirolide C (13-19diDesMetSPXC) and 1.2% of YTX were also detected in echinoderms, gastropods, crustaceans and bivalves (Figure 1A).

With regard to the concentrations reached, 13-desm SPXC and PnTXG showed maximum values of 20 and 63 µg kg^−1^, respectively, in bivalves (Figure 1B), specifically in *Magallana gigas* and *Tellina donacina*. However, the mean bivalve group level was 2 (±2) µg kg^−1^ for 13-desm SPXC and 5 (±6) µg kg^−1^ for PnTXG. For OA, maximum concentrations were detected in the crustacean *Inachus phalangium* (334 µg kg^−1^) and in an unidentified polychaete (332 µg kg^−1^). The crustacean mean was 86 (±115) µg kg^−1^ and the polychaetes mean was 103 (±154) µg kg^−1^. For DTX2, maximum concentrations were also registered in the same unidentified polychaete, with 235 µg kg^−1^ and in the bivalve *M. galloprovincialis* with 181 µg kg^−1^ (bivalve group mean of 67 ± 40 µg kg^−1^). It is important to remark that only one out of nine polychaetes analyzed showed concentrations > limit of quantification (LOQ). For YTX, maximum concentrations were recorded in *Neptunea contraria* (118 µg kg^−1^), the only gastropod out of the 191 analyzed showing levels above the LOQ, in the bivalve *M. galloprovincialis* (104 µg kg^−1^, mean of 89 ± 11 µg kg^−1^) and in *Necora puber* (83 µg kg^−1^), the unique crustacean out of the 83 analyzed with levels above the LOQ. Finally, for 13-19diDesMetSPXC, the highest concentration (2 µg kg^−1^) was detected in the crustacean *Macropipus tuberculatus*. No AZAs, pectenotoxin 2 (PTX2), DTX1, GYM A, other YTXs or 20 methyl spirolide G (20-met SPXG) concentrations above the LOD were found in any of the samples tested.

### 2.2. Mussels versus Different Groups of Marine Invertebrates

**Figure 2:**
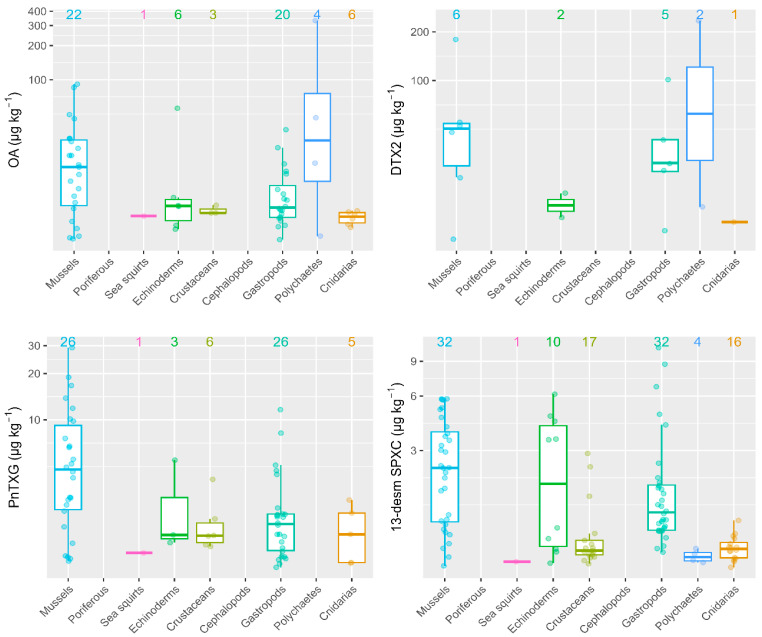
Toxin concentrations in mussels (*M. galloprovincialis*) and other invertebrate groups sampled on the same date and location. Figures at the top indicate the number of observations where toxins were detected.

In general, *M. galloprovincialis* were the organisms that attained higher toxin concentrations as compared to other invertebrate groups sampled on the same date and identical location. Regarding the four toxins that showed the highest prevalence and concentrations (OA, DTX2, PnTXG and 13-desm SPXC), mussels always showed the highest average level concentrations, except in a single particular case (Figure 2). Specifically, only one sample of an unidentified polychaete exceeded the OA and DTX2 concentrations achieved by mussels, showing values of 332 and 235 µg kg^−1^, respectively. For PnTXG, mussels were the organisms with the highest average toxin levels. Finally, for 13-desm SPXC, three out of four gastropod species evaluated (*Nassarius* sp., *Patella* sp., *Monodonta lineata* and *Haliotis tuberculata*) and one echinoderm sample (*Asterina* sp.) showed mean 13-desm SPXC concentrations slightly higher than those in mussels, with values of 11, 9, 7 and 6 µg kg^−1^, respectively (Figure 2).

### 2.3. Correlation between Mussels and Different Groups of Marine Invertebrates

**Figure 3:**
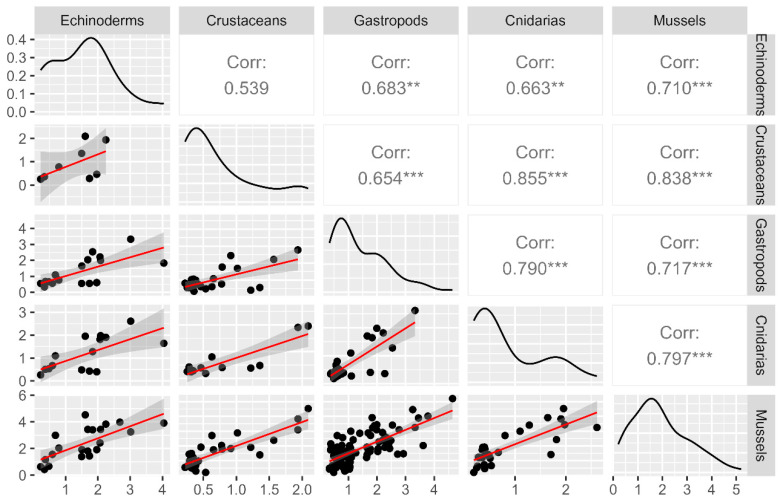
Correlations and regressions between the mean LTs concentrations in mussels (*M. galloprovincialis*) and four groups of marine invertebrates (echinoderms, crustaceans, gastropods, and cnidarians) sampled on the same date and location. The data have been logarithmically transformed. The correlation coefficients with significant levels are ** *p* < 0.05, and *** *p* < 0.01.

Positive correlations between the mean LTs concentrations in mussels and four different invertebrate groups (those with a number of more than ten observations concurrently with mussels: echinoderms, crustaceans, gastropods, and cnidarians) were obtained. The most significant correlations were found with crustaceans (R = 0.838) and the lowest, but still significant, with echinoderms (R = 0.710) (Figure 3).

## 3. Discussion

This study ran a pioneer comprehensive screening of multiple LTs in traditional and non-traditional invertebrate vectors in NW Spain (1150 samples belonging to different species of nine marine invertebrate groups). The CIs PnTXG (12.5–25%) and 13-desm SPXC (20.5–75%), were the two toxins with the highest prevalence, not only in bivalves but also in most of the studied groups (gastropods, crustaceans, cnidarians, echinoderms, cephalopods, and sea squirts). The results demonstrate that these compounds can be found in a broader variety of marine invertebrates than previously known.

The prevalence of CIs in bivalves worldwide was documented in other studies. For instance, Davidson et al. (2015) [9] reported numerous references regarding their presence in a wide variety of bivalve species. Amzil et al. (2023) [50] declared that, since January, 2018 unregulated LTs (SPXs, PnTXs among others) have been quantified every year in French shellfish (various species of bivalve and whelks as representative of gastropods). In Rambla-Alegre et al. (2018), CIs were detected in 69% of the mussel (*M. galloprovincialis* and *M. edulis*) samples, in 29% of the oysters and in 24% of the clams analyzed. The frequency of detection depends on the CIs group: 13-desm SPXC was detected more frequently in oysters (23%) followed by mussels (21%). However, PnTXG was more often detected in mussels (61%) and clams (23%) [10]. The presence of CIs in invertebrates other than bivalves has only been documented in a few studies. Among them, Kvrgić et al. (2021) did not detect CIs in the sea squirt *Microcosmus* spp. from the northern Adriatic Sea [11], whereas Silva et al. (2013) [47] indicated for the first time the presence of 13-desm SPXC in four gastropods (*Gibbula umbilicalis*, *Nucella lapillus*, *Monodonta* sp., *Patella intermedia*) and one echinoderm (*Marthasterias glacialis*). The present study is the first document worldwide to report the presence of PnTXG and 13-desm SPXC in cephalopods, crustaceans, sea squirts, and polychaetes (for the latter only 13-desm SPXC).

OA was the second LT with the biggest prevalence. The values detected in polychaetes (44%) and sea squirts (25%) are quite high but the number of samples analyzed in these groups was too low (n = 9 and 4, respectively) to draw robust conclusions. A review of the presence of OA and other phycotoxins from the phylum Annelida was recently published [51]. In our study, OA was found in two species different from those previously reported, *Aphrodita aculeata* and *Sipunculus nudus*. To the best of our knowledge, this is the first report of OA in sea squirts. OA was also detected in bivalves, crustaceans, gastropods, and echinoderms but in lower percentages (below 14%). The presence of this toxin in species belonging to these groups has been previously reported [37,38,42,43,47,52].

The prevalence of the other LTs detected, namely DTX2, YTX, and 13-19 diDesMetSPXC did not exceed 3.9% except for DTX2 in polychaetes (11.2%). This is also the first time DTXs are reported in polychaetes.

Despite their high prevalence, the mean concentrations obtained for 13-desm SPXC and PnTXG were low, below 2 (±2) µg kg^−1^ and 5 (±6) µg kg^−1^, respectively, in bivalves, the group with the highest concentrations. Only two species showed upper maximum values, *M. gigas* (21 µg kg^−1^ of 13-desm SPXC) and *T. donacina* (63 µg kg^−1^ of PnTXG). SPXs and PnTXs levels reported for bivalves (and other shellfish) all over the world vary widely. In the first report of SPXs in Galicia [22], similar 13-desm SPXC concentrations (ranging from 13 to 20 µg kg^−1^) were detected in mussels (*M. galloprovincialis*), but levels <LOQ were found in razor clams (*Ensis arcuatus*)*,* while in clams it was not detected. Moreover, Blanco et al., 2023 reported that more than 75% of the 13-desm SPXC concentrations in the monitoring program of Galicia between 2014 and 2021 were below 10 µg kg^−1^, with maximum values found in raft mussels (*M. galloprovincialis*), slightly below 80 µg kg^−1^ [20]. In other Atlantic coast countries, for instance in Arcachon Bay (France), the maximum 13-desm SPXC levels during the 2005 monitoring program were 47 µg kg^−1^ in oysters [53], while in Norway the total amount of estimated SPXs in mussels (*M. edulis*) was 103 µg kg^−1^ [54]. For PnTXs, the highest concentrations found in wild (intertidal) mussels (*M. galloprovincialis*) from the Spanish Atlantic and Cantabrian coasts did not exceed 15 µg·kg^−1^, and were for most samples below 3 µg·kg^−1^ [17]. Concentrations of PnTXG and 13-desm SPXC, ranging from 1.8 to 3.1 µg·kg^−1^ and 1.2 to 6.9 µg·kg^−1^, respectively, were also detected on the Atlantic coast of Galicia [12]. Rambla-Alegre et al. (2018) analyzed 96 samples from different European areas and detected CIs in 52% of them at low levels from 0.1 to 12 µg·kg^−1^ for PnTXG and 26–66 µg·kg^−1^ for 13-desm SPXC. In their survey, surf clams and blue mussels (*M. edulis*) were the fresh samples with the highest values for 13-desm SPXC (63 µg·kg^−1^) and PnTXG (5.1 µg·kg^−1^), respectively [10]. The highest concentration of PnTXG ever reported was found in mussels (*M. galloprovincialis*) from the Ingril lagoon, in the French Mediterranean, with up to 1244 µg·kg^−1^, and base levels above 40 µg·kg^−1^ of PnTXG [55].

With regard to OA, it should be noted that, although the mean concentration detected in polychaetes was moderate (103 µg·kg^−1^), an unidentified polychaete registered OA levels above the EU legal limit (332 µg·kg^−1^) and also DTX2 concentrations of 235 µg kg^−1^. Nevertheless, this specimen was the only one among the polychaetes analyzed with OA results above the EU legal limit. As indicated by Pires et al. (2023) [51], quick OA accumulation in polychaete tissues has been previously reported for *Laeonereis* sp. (maximum = 164.5 µg total OA kg^−1^) [43] and *Sabella spallanzanii* (max. 37 µg kg^−1^) [40] during mid- and late-bloom stages of *Dinophysis acuminata* complex dinoflagellates. However, these concentrations were significantly lower than those found in the present study. Also, rapid accumulation of OA and, to a lesser extent, DTX1 was confirmed in *Laeonereis acuta* under controlled laboratory exposure to *Prorocentrum lima* at cell densities of 2·10^3^ to 2·10⁴ cell mL^−1^ [44]. As far as we know, there are no previous references to DTX2 accumulation in polychaetes. In crustaceans, the average OA concentration recorded in this study was below the legal limit (86 µg kg^−1^) except for a maximum of 334 µg kg^−1^ in the crab *I. phalangium*. Several human-poisoning incidents due to the consumption of contaminated crustaceans have been reported worldwide, including in Europe [52]. In Portugal, a DSP intoxication was associated with the consumption of contaminated crabs (*Carcinus maenas*) containing 322 µg OA eq. kg^−1^ of edible tissue [37]. In Norway, several hundred people presented DSP symptoms after eating brown crabs (*Cancer pagurus*) [38]. In both cases, the toxin profile of the contaminated crabs was mostly composed (>90%) of esterified OA derivatives, which is in accordance with its predation predominantly on benthic shellfish (razor clams, clams, and cockles) rather than on mussels (*M. galloprovincialis*) (which usually present higher free OA percentages) [37]. So, although OA-esterified forms were not analyzed here, it is more than likely that the toxicities found in our study in *I. phalangium* are much higher than those previously detected in other species, suggesting that this species could represent an important vector for the transmission of these toxins in the food web. Finally, the YTX and 13–19 diDesMetSPXC concentrations detected in the present study were always very low.

In a recent study, Louzao et al. (2022) [56] propose that climate change could affect the prevalence of HABs and the impact of phycotoxins on human and ecosystem health. In this sense, Silva et al. (2013) [47] suggested that due to the detection of new vectors, particularly those potentially used as food resources, the monitoring of marine toxins should be extended to species other than bivalves and even to new toxins. This agrees with García-Altares et al. (2014) [23] who suggested that emerging toxins as CIs should be included in the shellfish safety monitoring programs of LTs.

In areas such as Galicia (NW Spain) with about 1500 km of coast, direct control of all the potentially consumed species is impossible. Therefore, an efficient and safe monitoring program requires the use of sentinel species such as mussels, which provide key information about the toxin levels in a given area. Mussels conjugate several characteristics that make them effective bioindicators as filter-feeding behavior, long lifespan, sedentary nature, sensitivity to environmental changes, and wide distribution. By analyzing toxin levels in mussels, early warnings about the development of toxic episodes become available to guide decision makers and the implementation of protective measures. In this sense, our study demonstrated that, within the studied groups, mussels (*M. galloprovincialis*) are the invertebrates that generally presented the highest levels for the studied LTs in Galician samples. In general, the LTs concentration in mussels was highly correlated with the levels found in other invertebrates (echinoderms, crustaceans, gastropods, and cnidarians) in the same ecosystem, indicating that they could be good bioindicators of the concentrations of the studied toxins in their environment. In this sense, Hess et al., 2013 [55] reported that mussels (*M. galloprovincialis*) were always more contaminated with PnTXs than clams and could be used as sentinel species. Therefore, monitoring the concentration of these compounds in mussels constitutes: (i) an effective indicator of the overall health and safety of marine invertebrates, and (ii) a source of valuable information for the management and regulation of some fishery and aquaculture industries. The polychaetes group might be an exception. However, the small number of samples in the present study precludes drawing solid conclusions and further surveys of this group should be envisaged.

## 4. Conclusions

The present work reported for the first time in NW Spain the presence of: (i) PnTXG and 13-desm SPXC in cephalopods, crustaceans, and sea squirts; (ii) 13-desm SPXC in polychaetes; (iii) OA in the polychaetes *A. aculeata* and *S. nudus* and in sea squirts; (iv) DTX2 in polychaetes. Overall, it can be stated that the low concentrations of CIs found suggest that health risks associated with SPXs and PnTXs through shellfish consumption are low. However, the results also suggest that human seafood consumers could be exposed to moderate levels of regulated toxins (mainly OA and DTX2) from a variety of non-traditional vectors during intense toxic outbreaks. The obtained results confirm that monitoring programs based on the use of mussels (at least, *M. galloprovincialis*) as sentinel organisms could be very effective for the correct management and regulation of a large majority of marine invertebrates, provided mussels and the non-traditional vectors are from the same sampling location. However, for polychaetes a more in-depth survey to gather representative results would be needed to raise such conclusion.

## 5. Materials and Methods

### 5.1. Sampling of Non-Traditional Invertebrate Vectors

The biological material included in the present study was obtained by means of samplings carried out in three different projects, described as follows.

#### 5.1.1. Toxemer

**Figure 4:**
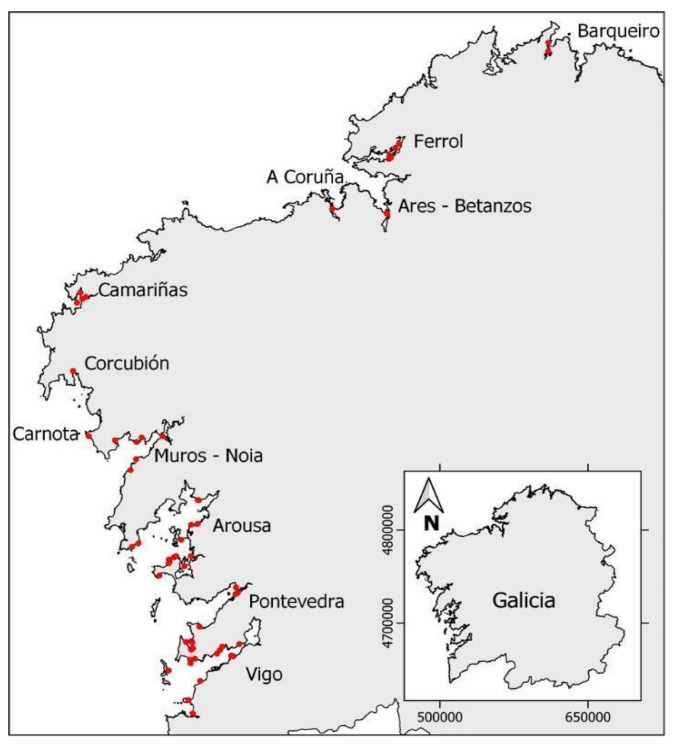
Sampling locations of the TOXEMER project framework. Red points indicate the approximate sampling point in each Ría. The inserted map shows the sampled area. Source [35].

The main scope of the TOXEMER project (Emerging Toxins in Galicia), was to update knowledge of the prevalence of new or emerging toxins in Galicia and to evaluate the risk posed by toxins or vectors not usually monitored by the marine environment control systems. Under this scope, a total of 1008 samples from different marine invertebrate species (including bivalves, echinoderms, gastropods, crustaceans, polychaetes, porifera, sea squirts, and cnidarians) were sampled from 64 different points along the Galician coast (NW Spain, Figure 4) from April 2021 to December 2022. The organisms were collected both, from the intertidal zone at low tide (manually) or from the subtidal zone (by free-diving or dredging).

#### 5.1.2. Primrose

**Figure 5:**
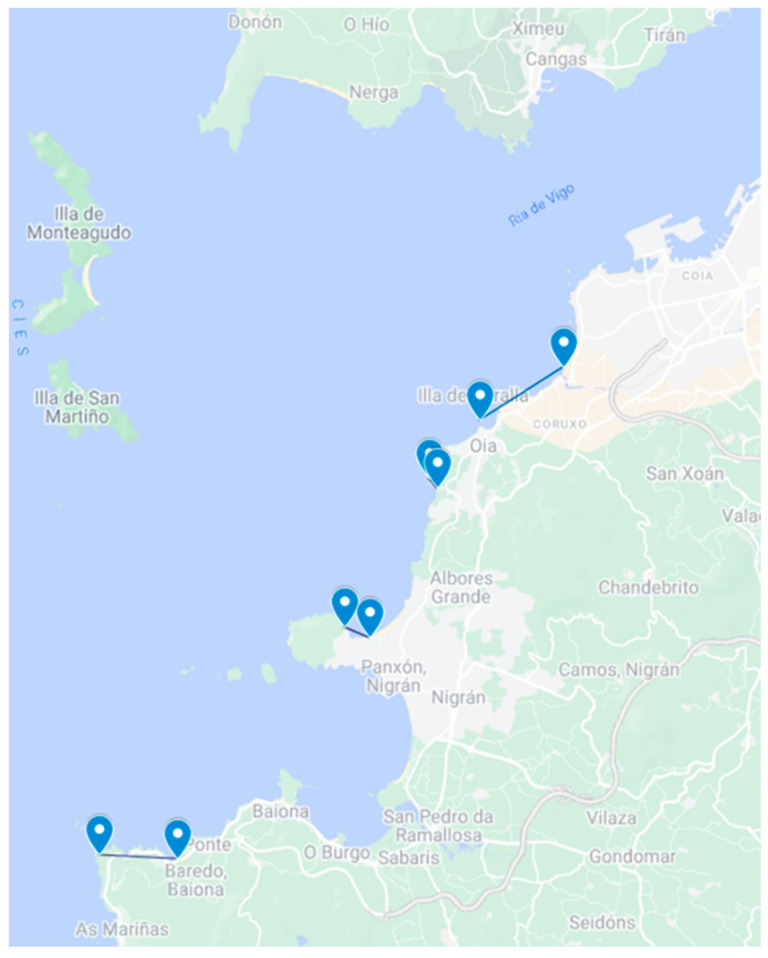
Sampling areas of the PRIMROSE Project in Ría de Vigo, 2021. Each area is between two consecutive points.

**Figure 6:**
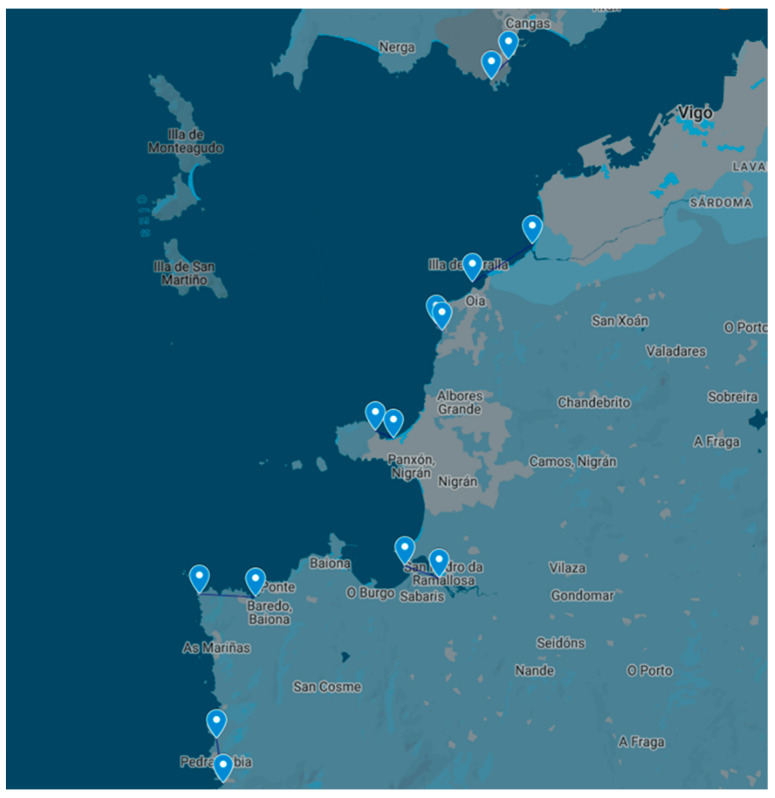
Sampling areas of the PRIMROSE Project in Ría de Vigo, 2022. Each area is between two consecutive points.

The PRIMROSE project (Predicting Risk and Impact of Harmful Events on the Aquaculture Sector), included samples from the rocky shores from the Ría de Vigo during spring–early autumn of 2021–2022. In 2021, four locations were sampled, while in 2022 three additional areas were included (Figure 5 and Figure 6). In these areas, we managed to obtain 117 samples from different species of marine invertebrates, including echinoderms, gastropods, crustaceans, and cnidarians. Whenever available, samples from wild mussels (*M. galloprovincialis*) were also collected, with the aim of comparing the toxin results in this sentinel species with those in non-traditional vectors.

#### 5.1.3. Descarsel

The DESCARSEL project runs an oceanographic survey every year using research vessels and fishery units from the IEO-CSIC or the Ministry of Agriculture, Fisheries and Food (MAPA). In September 2021, a survey was performed on board the Miguel Oliver Fishery Research Vessel. This survey took place along the Galician coast and samples were obtained by trawling fishing in 12 different stations from south (S) Ría de Vigo to N Ría de Muros-Noia (coordinates indicated in Table S1). A total of 25 samples of marine invertebrates were collected for the present work. The organisms belonged to the same groups as in the PRIMROSE project, plus tunicates, cephalopods, and polychaetes. Thus, DESCARSEL samplings provided a valuable source of biological material from diverse marine fauna and areas away from the shore.

Detailed information on every sample considered in the present study, including sampling date, location, and the taxonomic identification of the organisms, is provided in Supplementary Materials (Table S1).

### 5.2. Chemicals and Reagents

Chemicals were liquid chromatography-mass spectrometry (LC-MS) or high-performance liquid chromatography (HPLC) grade quality. For acidic conditions, mobile phases were prepared from LC-MS acetonitrile (MeCN) (LiChrosolv^®^, Supelco, Darmstadt, Germany), formic acid (VWR Chemicals, Leuven, Belgium), water (Biosolve Chimie, Dieuze, France), and ammonium formate (HPLC grade, FlukaTM, Seelze, Germany). For sample extraction, LC-MS methanol (MeOH) (LiChrosolv^®^, Supelco, Darmstadt, Germany) was used. For alkaline chromatographic conditions, MeCN (LC-MS grade, Scharlab, Sentmenat, Spain), MeOH (HPLC grade, VWR, Llinars del Vallés, Spain), and ammonium hydroxide (NH_4_OH, 25%), (Merck, Barcelona, Spain). A Milli-Q gradient system fed with an Elix Advantage-10 (Millipore Ibérica, Madrid, Spain) was the source of ultrapure water. Certified reference materials (CRMs) for purified toxin standards of AZAs (AZA1, AZA2, AZA3), OA, DTX1, DTX2, PTX2, YTX, homoyessotoxin (homoYTX), (13-desm SPXC, PnTXG, GYMA, and quality control standards (QCSs) from additional SPXs 13-19diDesMetSPXC and 20-methyl SPXG were from the Institute of Biotoxin Metrology (IBM), National Research Council Canada (NRCC, Halifax, NS, Canada) and from Laboratorio CIFGA S.A. (Lugo, Spain). Reference materials were used to prepare stock and working calibration solutions by dilution with LC-MS grade MeOH for external calibration purposes.

Additional CRMs, for quality control purposes, were NRC-CRM-FDMT1, CRM-DSP-MUS, CRM-AZA-MUS, and CRM-ZERO-MUS, all from the IBM_NRCC.

### 5.3. Sample Preparation

Raw samples were thoroughly cleaned outside with fresh water to remove sand and foreign material. For bivalve molluscs, samples were opened by cutting the adductor muscle, rinsed inside with fresh water, and the soft tissues separated from the shell. For other invertebrates, the tissue removal procedure was adapted to each species (i.e., for crabs, sea urchins, and gastropods the shell was broken if needed, for sea stars the arms were opened with a scalpel). The obtained tissues were placed in a sieve to remove salt water. Whenever possible a representative aliquot of pooled tissues was obtained and homogenized in a blender. Sub-samples from this homogenate were dispensed in plastic containers and immediately frozen and kept at −18 °C until analysis.

### 5.4. Extraction Procedure

An aliquot of tissue homogenate was accurately weighed (2.00 g ± 0.05 g) into a 50 mL polypropylene centrifuge tube (Eppendorf). Single or double extraction with 100% MeOH was conducted as indicated in [57]. Methanolic extracts were filtered through 0.22 µm PTFE or PES syringe filters into vials ready to be analyzed by liquid chromatography tandem quadrupole mass spectrometry (LC-MS/MS).

### 5.5. LC-MS/MS Analysis

#### 5.5.1. Acidic Chromatographic Conditions

LC-MS/MS analyses were conducted under acidic conditions following the EU-Harmonised Standard Operation Procedure (SOP) [57]. An AB SCIEX (Redwood City, CA, USA) 4500 MS/MS coupled to an Agilent (Manchester, UK) 1260 UHPLC was used for analysis. Toxins were separated in an XBridge^TM^ C18 column, 50 mm (length) × 2.1 mm (id), 2.5 μm particle size. The mobile phase consisted of 100% water containing 2 mM ammonium formate and 50 mM formic acid in channel A, and acetonitrile:water (95:5, *v*:*v*) containing 2 mM ammonium formate and 50 mM formic acid in channel B. Other chromatographic conditions are as indicated in Table 1a. Mass spectrometer source conditions are as in Table 1b. Multiple Reaction Monitoring (MRM) conditions and LOQs are specified in Tables S2 and S3, respectively.

Data acquisition and processing were performed using the Software Analyst 1.6.2, ABSCIEX Multiquant 3.0.2. An external standard calibration procedure with six calibration levels/compounds was used to determine LTs concentration in the samples. For quality control purposes, the four CRMs mentioned in the chemicals and reagents section were analyzed with each sample batch. In addition, the quality control criteria for acceptance of the quantitative results during the analyses of lipophilic marine Biotoxins by LC-MS/MS, as specified in the EU-Harmonised SOP, 2015 [57] were also checked.

#### 5.5.2. Alkaline Chromatographic Conditions

The analyses have been carried out on an Exion LC AD™ System (SCIEX, Framingham, MA, USA) coupled to a Qtrap 6500+ mass spectrometer (SCIEX) through an IonDrive Turbo V interface in electrospray mode according to Rossignoli et al., 2021 [58] with slight modifications. Gemini NX C18 column 50 mm (length) × 2 mm (id), 3 µm (particle size) from Phenomenex (Torrance, CA, USA) was used to separate toxins. Mobile phase A was water and B MeCN 90%, both containing 6.7 mM NH_4_OH (pH = 11). Other chromatographic conditions as indicated in Table 2a. The mass spectrometer parameters optimized using toxin standards are indicated in Table 2b. For specific MS/MS fragmentation conditions, collision energies for all the toxins validated, LODs (s/n = 3) and LOQs (s/n = 10) check the previously published method [58]. Data acquisition and processing were performed using the Sciex OS Software, AB SCIEX version 3.0.0.3339, and as for acidic chromatographic conditions, an external standard calibration procedure was used, with six calibration levels/compound.

### 5.6. Statistical Analyses

Statistical analyses, graphs, and correlation coefficients—for comparison between mussels (*M. galloprovincialis*) and other invertebrate groups—were carried out with R [59]. LODs used to calculate prevalences were those of the alkaline chromatographic method (the most sensitive). Although this may lead to a slight overestimation of the prevalence percentages achieved, it does not imply a substantial modification of the results obtained.

## Figures and Tables

**Table 1:** (a) Acidic chromatographic conditions. (b) Mass spectrometer parameters for acidic chromatographic conditions.

(a)			
**Flow: [0.25–0.40] mL/min**			
**Injection Volume: 5 µL, Injector Temp: 4 °C** ** ** **Column Temp: 30 °C**			
**Time (min)**	**Flow**	**%A**	**%B**
0.10	0.25	90	10
6.50	0.25	20	80
8.50	0.25	0	100
9.61	0.25	0	100
10.00	0.40	0	100
11.50	0.40	0	100
11.60	0.25	90	10
15.00	0.25	90	10
(b)			
**Parameters**		**Positive Mode**	**Negative Mode**
Curtain Gas (CUR)		25	25
Collision Gas (CAD)		High	High
Voltage (IS) V		4500	−4500
Temperature (TEMP) °C		500	500
Gas 1 (GS1) psi		50	50
Gas 2 (GS2) psi		50	50

**Table 2:** (a) Alkaline chromatographic conditions. (b) Mass spectrometer parameters for alkaline chromatographic conditions.

(a)			
**Flow: 0.40 mL/min**			
**Injection Volume: 1 µL, Injector Temp: 4 °C** ** ** **Column Temp: 40 °C**			
**Time (min)**	**Flow**	**%A**	**%B**
0.50	0.40	78	22
3.85	0.40	5	95
6.25	0.40	5	95
6.75	0.40	78	22
8.75	0.40	78	22
(b)			
**Parameters**		**Positive Mode**	**Negative Mode**
Curtain Gas (CUR)		30	30
Collision Gas (CAD)		Medium	Medium
Voltage (IS) V		5000	−4500
Temperature (TEMP) °C		600	600
Gas 1 (GS1) psi		75	75
Gas 2 (GS2) psi		75	75

## Data Availability

Data are available on request in the Centro de Investigacións Mariñas.

## Supplementary Materials

The following supporting information can be downloaded at: https://www.mdpi.com/article/10.3390/toxins15110631/s1, Table S1: Date, sampling location, Ría, class, and species analyzed in the study between 2021 to 2022; Table S2: Multiple Reaction Monitoring (MRM) conditions for lipophilic toxins (LTs) determination. Precursor ion Q1 = *m*/*z* ratio in the first quadrupole, Product ion Q3 = *m*/*z* ratio in the third quadrupole, RT (min) = retention time, DP(v) = declustering potential, EP(v) = entrance potential, CE(v) = collision energy, and CXP(v) = collision cell exit potential, Table S3: Limits of quantification (LOQs) for LTs determination with acidic chromatographic conditions.
