# Supplementary material for: Lipophilic Shellfish Poisoning Toxins in Marine Invertebrates from the Galician Coast
Source: Toxins (Basel). 2023 Oct 27;15(11):631. doi: 10.3390/toxins15110631 (PMC10675701; doi:10.3390/toxins15110631)
Supplement: Supplementary file 1 [file toxins-15-00631-s001.zip › toxins-2634302-supplementary.pdf]

Supplementary material: Lipophilic shellfish poisoning toxins in marine invertebrates from the Galician coast

Table S1. Date, sampling location, Ría, class, and species analyzed in the study between 2021 to 2022.

Date	Sampling location	Ria	Class	Species
May 11, 2021	Ferrol. As Pías	Ferrol	Bivalves	*Modiolus modiolus*
May 11, 2021	Ferrol. As Pías	Ferrol	Bivalves	*Acanthocardia tuberculata*
May 25, 2021	Vilanova de Arousa. Corón	Arousa	Bivalves	*Ensis siliqua*
May 25, 2021	Vilanova de Arousa. Corón	Arousa	Bivalves	*Ruditapes philippinarum*
May 25, 2021	Vilanova de Arousa. Corón	Arousa	Bivalves	*Cerastoderma edule*
May 25, 2021	Vilanova de Arousa. Corón	Arousa	Bivalves	*Mytilus galloprovincialis*
May 25, 2021	Vilanova de Arousa. Corón	Arousa	Crustaceans	*Polybius sp.*
May 25, 2021	Vilanova de Arousa. Corón	Arousa	Crustaceans	*Polybius sp.*
May 25, 2021	Vilanova de Arousa. Corón	Arousa	Gastropods	*Ocenebra sp.*
May 25, 2021	Lira. Ximpron	Corcubión	Echinoderms	*n.i.*
July 26, 2021	Vilanova de Arousa. Corón	Arousa	Cnidarias	*n.i.*
July 26, 2021	Vilanova de Arousa. Corón	Arousa	Gastropods	*Gibbula sp.*
July 26, 2021	Vilanova de Arousa. Corón	Arousa	Bivalves	*Mytilus galloprovincialis*
July 26, 2021	Vilanova de Arousa. Corón	Arousa	Gastropods	*Nucella sp.*
July 26, 2021	Vilanova de Arousa. Corón	Arousa	Gastropods	*Littorina sp.*
July 26, 2021	Vilanova de Arousa. Corón	Arousa	Gastropods	*Patella sp.*
August 5, 2021	Rianxo. Porrón	Arousa	Gastropods	*Littorina sp.*
August 5, 2021	Rianxo. Porrón	Arousa	Crustaceans	*Polybius sp.*
August 5, 2021	Rianxo. Porrón	Arousa	Bivalves	*Mytilus galloprovincialis*
August 6, 2021	Vilanova de Arousa. Corón	Arousa	Gastropods	*Nucella sp.*
August 6, 2021	Vilanova de Arousa. Corón	Arousa	Bivalves	*Ruditapes philippinarum*
August 6, 2021	Vilanova de Arousa. Corón	Arousa	Bivalves	*Cerastoderma edule*
August 6, 2021	Vilanova de Arousa. Corón	Arousa	Bivalves	*Mytilus galloprovincialis*

August 6, 2021	Vilanova de Arousa. Corón	Arousa	Gastropods	*Littorina sp.*
August 6, 2021	Vilanova de Arousa. Corón	Arousa	Gastropods	*Littorina sp.*
August 6, 2021	Vilanova de Arousa. Corón	Arousa	Cnidarias	*n.i.*
August 6, 2021	Vilanova de Arousa. Corón	Arousa	Crustaceans	*Balanus sp.*
August 6, 2021	Vilanova de Arousa. Corón	Arousa	Gastropods	*Patella sp.*
August 20, 2021	Vilanova de Arousa. Corón	Arousa	Crustaceans	*Balanus sp.*
August 20, 2021	Vilanova de Arousa. Corón	Arousa	Gastropods	*Littorina sp.*
August 20, 2021	Vilanova de Arousa. Corón	Arousa	Gastropods	*Littorina sp.*
August 20, 2021	Vilanova de Arousa. Corón	Arousa	Gastropods	*Nucella sp.*
August 20, 2021	Vilanova de Arousa. Corón	Arousa	Cnidarias	*n.i.*
August 20, 2021	Vilanova de Arousa. Corón	Arousa	Bivalves	*Mytilus galloprovincialis*
August 20, 2021	Vilanova de Arousa. Corón	Arousa	Gastropods	*Patella sp.*
August 9, 2021	Nerga. Os Castros	Vigo	Gastropods	*Patella sp.*
August 9, 2021	Nerga. Os Castros	Vigo	Echinoderms	*n.i.*
August 9, 2021	Nerga. Os Castros	Vigo	Cnidarias	*n.i.*
August 9, 2021	Nerga. Os Castros	Vigo	Gastropods	*Littorina sp.*
August 10, 2021	Aldán. Vilariño	Pontevedra	Gastropods	*Patella sp.*
August 10, 2021	Aldán. Vilariño	Pontevedra	Cnidarias	*n.i.*
August 10, 2021	Aldán. Vilariño	Pontevedra	Gastropods	*Littorina sp.*
August 25, 2021	Bueu. Beluso	Pontevedra	Echinoderms	*n.i.*
August 25, 2021	Bueu. Beluso	Pontevedra	Gastropods	*Nucella sp.*
August 25, 2021	Bueu. Beluso	Pontevedra	Gastropods	*Littorina sp.*
August 25, 2021	Bueu. Beluso	Pontevedra	Gastropods	*Crepidula sp. fornicata*
August 25, 2021	Bueu. Beluso	Pontevedra	Echinoderms	*n.i.*
August 25, 2021	Bueu. Beluso	Pontevedra	Cnidarias	*n.i.*
August 25, 2021	Bueu. Beluso	Pontevedra	Gastropods	*Patella sp.*
August 25, 2021	Bueu. Beluso	Pontevedra	Bivalves	*Mytilus galloprovincialis*
September 23, 2021	Vilanova de Arousa. Corón	Arousa	Gastropods	*Monodonta sp.*
September 23, 2021	Vilanova de Arousa. Corón	Arousa	Gastropods	*Gibbula sp.*

September 23, 2021	Vilanova de Arousa. Corón	Arousa	Gastropods	*Nucella sp.*
September 23, 2021	Vilanova de Arousa. Corón	Arousa	Gastropods	*Littorina sp.*
September 23, 2021	Vilanova de Arousa. Corón	Arousa	Cnidarias	*n.i.*
September 23, 2021	Vilanova de Arousa. Corón	Arousa	Gastropods	*Patella sp.*
September 23, 2021	Vilanova de Arousa. Corón	Arousa	Bivalves	*Mytilus galloprovincialis*
May 26, 2021	Muros I. Esteiro	Muros-Noia	Gastropods	*Patella sp.*
September 7, 2021	Rianxo. Porrón	Arousa	Bivalves	*Mytilus galloprovincialis*
September 24, 2021	Rianxo. Salto do ladrón	Arousa	Gastropods	*Littorina sp.*
September 24, 2021	Rianxo. Salto do ladrón	Arousa	Bivalves	*Mytilus galloprovincialis*
September 24, 2021	Rianxo. Salto do ladrón	Arousa	Gastropods	*n.i.*
June 29, 2021	Barallobre	Ferrol	Bivalves	*Mytilus galloprovincialis*
June 29, 2021	Miño	Betanzos	Bivalves	*Ruditapes philippinarum*
June 29, 2021	Pasaxe. Sta Cristina	A Coruña	Bivalves	*Cerastoderma edule*
June 29, 2021	Camariñas	Camariñas	Bivalves	*Mytilus galloprovincialis*
June 30, 2021	Corcubión. Pr.Cee	Corcubión	Bivalves	*Mytilus galloprovincialis*
June 30, 2021	Barqueiro	Barqueiro	Bivalves	*Cerastoderma edule*
July 2, 2021	Arousa I. Meloxo	Arousa	Bivalves	*Venerupis pullastra*
July 2, 2021	Arousa VI	Arousa	Bivalves	*Venerupis pullastra*
July 2, 2021	Muros I. Abelleira	Muros-Noia	Bivalves	*Cerastoderma edule*
July 5, 2021	Muros I	Muros-Noia	Bivalves	*Cerastoderma edule*
July 6, 2021	Barqueiro. San Fiz	Barqueiro	Bivalves	*Magallana gigas*
July 6, 2021	Miño. Lombo Espiñeira	Betanzos	Bivalves	*Ruditapes philippinarum*
July 6, 2021	Pasaxe. Sta Cristina	A Coruña	Bivalves	*Cerastoderma edule*
July 6, 2021	Camariñas. Enseada Vasa	Camariñas	Bivalves	*Mytilus galloprovincialis*
July 6, 2021	Arousa VI. Sarrido	Arousa	Bivalves	*Ruditapes philippinarum*
October 29, 2021	Arousa	Arousa	Polychaetes	*Aphrodita aculeata*
July 8, 2021	Barallobre. Rampa	Ferrol	Bivalves	*Mytilus galloprovincialis*
July 7, 2021	Corcubión. Pr.Cee	Corcubión	Bivalves	*Cerastoderma edule*
July 7, 2021	Arousa I. Meloxo	Arousa	Bivalves	*Venerupis pullastra*

July 14, 2021	Barqueiro. Pr. Salgueira	Barqueiro	Bivalves	*Mytilus galloprovincialis*
July 13, 2021	Barallobre. Rampa	Ferrol	Bivalves	*Mytilus galloprovincialis*
July 13, 2021	Miño. Lombo Espiñeira	Betanzos	Bivalves	*Ruditapes philippinarum*
July 13, 2021	Pasaxe. Sta Cristina	A Coruña	Bivalves	*Cerastoderma edule*
July 13, 2021	Camariñas. Enseada Vasa	Camariñas	Bivalves	*Mytilus galloprovincialis*
July 13, 2021	Corcubión. Pr.Cee	Corcubión	Bivalves	*Mytilus galloprovincialis*
July 15, 2021	Camariñas. Rio da Ponte	Camariñas	Bivalves	*Ruditapes decussatus*
July 20, 2021	Barqueiro. Pr. Salgueira	Barqueiro	Bivalves	*Mytilus galloprovincialis*
July 21, 2021	Miño. Lombo Espiñeira	Betanzos	Bivalves	*Ruditapes philippinarum*
July 20, 2021	Pasaxe. Sta Cristina	A Coruña	Bivalves	*Cerastoderma edule*
July 21, 2021	Camariñas. Rio da Ponte	Camariñas	Bivalves	*Ruditapes decussatus*
July 21, 2021	Corcubión. Pr.Cee	Corcubión	Bivalves	*Mytilus galloprovincialis*
July 23, 2021	Muros I. Abelleira	Muros-Noia	Bivalves	*Cerastoderma edule*
July 27, 2021	Barqueiro. Pr. Salgueira	Barqueiro	Bivalves	*Mytilus galloprovincialis*
July 27, 2021	Miño. Lombo Espiñeira	Betanzos	Bivalves	*Ruditapes philippinarum*
July 27, 2021	Pasaxe. Sta Cristina	A Coruña	Bivalves	*Cerastoderma edule*
July 27, 2021	Camariñas	Camariñas	Bivalves	*Ruditapes decussatus*
July 29, 2021	Corcubión. Pr.Cee	Corcubión	Bivalves	*Mytilus galloprovincialis*
July 27, 2021	Muros I. Abelleira	Muros-Noia	Bivalves	*Cerastoderma edule*
July 27, 2021	Muros III. Testal	Muros-Noia	Bivalves	*Cerastoderma edule*
July 29, 2021	Pont II. Niño do Corvo	Pontevedra	Bivalves	*Ensis ensis*
July 28, 2021	Pont V. Placeres	Pontevedra	Bivalves	*Ruditapes philippinarum*
July 29, 2021	Vigo I. Cies	Vigo	Bivalves	*Venerupis rhomboides*
July 29, 2021	Vigo II.2 Xunqueira	Vigo	Bivalves	*Ruditapes philippinarum*
August 3, 2021	Barqueiro. San Fiz	Barqueiro	Bivalves	*Mytilus galloprovincialis*
August 6, 2021	Barallobre. Rampa	Ferrol	Bivalves	*Mytilus galloprovincialis*
August 4, 2021	Miño. Lombo Espiñeira	Betanzos	Bivalves	*Venerupis pullastra*
August 6, 2021	Camariñas. Enseada Vasa	Camariñas	Bivalves	*Ruditapes decussatus*
August 3, 2021	Corcubión. Pr.Cee	Corcubión	Bivalves	*Mytilus galloprovincialis*

August 5, 2021	Pont II. San Cibrao	Pontevedra	Bivalves	*Ruditapes philippinarum*
August 3, 2021	Pont V. Campelo	Pontevedra	Bivalves	*Ruditapes philippinarum*
August 4, 2021	Vigo I. Cies	Vigo	Bivalves	*Venerupis rhomboides*
August 3, 2021	Vigo II.2 Xunqueira	Vigo	Bivalves	*Ruditapes philippinarum*
August 11, 2021	Barqueiro. Pr. Salgueira	Barqueiro	Bivalves	*Mytilus galloprovincialis*
August 10, 2021	Barallobre. Rampa	Ferrol	Bivalves	*Mytilus galloprovincialis*
August 10, 2021	Miño. Lombo Espiñeira	Betanzos	Bivalves	*Ruditapes philippinarum*
August 10, 2021	Pasaxe. Sta Cristina	A Coruña	Bivalves	*Cerastoderma edule*
August 10, 2021	Camariñas. Rio da Ponte	Camariñas	Bivalves	*Ruditapes decussatus*
August 11, 2021	Muros I. Abelleira	Muros-Noia	Bivalves	*Cerastoderma edule*
August 18, 2021	Barqueiro. Pr. Salgueira	Barqueiro	Bivalves	*Mytilus galloprovincialis*
August 19, 2021	Barallobre. Rampa	Ferrol	Bivalves	*Mytilus galloprovincialis*
August 18, 2021	Miño. Lombo Espiñeira	Betanzos	Bivalves	*Ruditapes philippinarum*
August 17, 2021	Pasaxe. Sta Cristina	A Coruña	Bivalves	*Cerastoderma edule*
August 18, 2021	Camariñas. Rio da Ponte	Camariñas	Bivalves	*Ruditapes decussatus*
August 17, 2021	Corcubión. Pr.Cee	Corcubión	Bivalves	*Cerastoderma edule*
August 18, 2021	Muros I	Muros-Noia	Bivalves	*Cerastoderma edule*
August 24, 2021	Barallobre	Ferrol	Bivalves	*Mytilus galloprovincialis*
August 25, 2021	Miño. Lombo Espiñeira	Betanzos	Bivalves	*Ruditapes philippinarum*
August 24, 2021	Pasaxe. Sta Cristina	A Coruña	Bivalves	*Cerastoderma edule*
August 24, 2021	Camariñas. Rio da Ponte	Camariñas	Bivalves	*Ruditapes decussatus*
August 25, 2021	Corcubión	Corcubión	Bivalves	*Cerastoderma edule*
August 24, 2021	Muros I	Muros-Noia	Bivalves	*Cerastoderma edule*
August 26, 2021	Arousa I. Meloxo	Arousa	Bivalves	*Venerupis pullastra*
August 23, 2021	Arousa VI	Arousa	Bivalves	*Ruditapes philippinarum*
August 23, 2021	Pont II. Aldán	Pontevedra	Bivalves	*Venerupis rhomboides*
August 25, 2021	Pont V. Placeres	Pontevedra	Bivalves	*Ruditapes philippinarum*
August 24, 2021	Vigo II.2	Vigo	Bivalves	*Ruditapes philippinarum*
September 1, 2021	Barallobre. Rampa	Ferrol	Bivalves	*Mytilus galloprovincialis*

August 31, 2021	Miño. Lombo Espiñeira	Betanzos	Bivalves	*Ruditapes philippinarum*
August 31, 2021	Pasaxe. Sta Cristina	A Coruña	Bivalves	*Cerastoderma edule*
September 3, 2021	Camariñas. Rio da Ponte	Camariñas	Bivalves	*Ruditapes philippinarum*
August 31, 2021	Corcubión. Pr.Cee	Corcubión	Bivalves	*Cerastoderma edule*
August 31, 2021	Muros I	Muros-Noia	Bivalves	*Cerastoderma edule*
August 31, 2021	Pont II. Area Brava	Pontevedra	Bivalves	*Venerupis rhomboides*
September 3, 2021	Pont V	Pontevedra	Bivalves	*Ruditapes philippinarum*
November 7, 2021	Rianxo. Porrón	Arousa	Crustaceans	*Polybius sp.*
November 7, 2021	Rianxo. Porrón	Arousa	Bivalves	*Cerastoderma edule*
November 7, 2021	Rianxo. Porrón	Arousa	Echinoderms	*Asterina sp.*
November 7, 2021	Rianxo. Porrón	Arousa	Bivalves	*Mytilus galloprovincialis*
November 7, 2021	Rianxo. Porrón	Arousa	Gastropods	*n.i.*
November 7, 2021	Rianxo. Porrón	Arousa	Poriferous	*n.i.*
November 7, 2021	Rianxo. Porrón	Arousa	Crustaceans	*n.i.*
November 5, 2021	Ramallosa. Esteiro Foz	Vigo	Bivalves	*Ruditapes philippinarum*
November 5, 2021	Ramallosa. Esteiro Foz	Vigo	Gastropods	*Littorina sp.*
November 5, 2021	Ramallosa. Esteiro Foz	Vigo	Gastropods	*Patella sp.*
November 5, 2021	Ramallosa. Esteiro Foz	Vigo	Bivalves	*Mytilus galloprovincialis*
November 5, 2021	Ramallosa. Esteiro Foz	Vigo	Cnidarias	*n.i.*
November 5, 2021	Ramallosa. Esteiro Foz	Vigo	Bivalves	*Ostrea edulis*
November 8, 2021	Porto do Son. Arnela	Muros-Noia	Gastropods	*Monodonta sp.*
November 8, 2021	Porto do Son. Arnela	Muros-Noia	Gastropods	*Gibbula sp.*
November 8, 2021	Porto do Son. Arnela	Muros-Noia	Gastropods	*Nucella sp.*
November 8, 2021	Porto do Son. Arnela	Muros-Noia	Polychaetes	*n.i.*
November 8, 2021	Porto do Son. Arnela	Muros-Noia	Echinoderms	*Asterina sp.*
November 8, 2021	Porto do Son. Arnela	Muros-Noia	Cnidarias	*n.i.*
November 8, 2021	Porto do Son. Arnela	Muros-Noia	Gastropods	*Patella sp.*
November 8, 2021	Porto do Son. Arnela	Muros-Noia	Gastropods	*Patella sp.*
November 8, 2021	Porto do Son. Arnela	Muros-Noia	Gastropods	*Patella sp.*

November 8, 2021	Porto do Son. Arnela	Muros-Noia	Bivalves	*Mytilus galloprovincialis*
November 8, 2021	Porto do Son. Arnela	Muros-Noia	Bivalves	*Mytilus galloprovincialis*
November 8, 2021	Pont V. Placeres	Pontevedra	Gastropods	*Littorina sp.*
November 8, 2021	Pont V. Placeres	Pontevedra	Gastropods	*Patella sp.*
November 8, 2021	Pont V. Placeres	Pontevedra	Crustaceans	*Polybius sp.*
November 8, 2021	Pont V. Placeres	Pontevedra	Bivalves	*Ostrea edulis*
November 8, 2021	Pont V. Placeres	Pontevedra	Bivalves	*Ruditapes philippinarum*
November 8, 2021	Pont V. Placeres	Pontevedra	Crustaceans	*Balanus sp.*
November 8, 2021	Pont V. Placeres	Pontevedra	Bivalves	*Cerastoderma edule*
November 8, 2021	Pont V. Placeres	Pontevedra	Bivalves	*Mytilus galloprovincialis*
September 1, 2021	Arousa. Bohido	Arousa	Bivalves	*Tellina donacina*
September 1, 2021	Arousa. Bohido	Arousa	Echinoderms	*Leptosynapta sp.*
September 1, 2021	Arousa. Bohido	Arousa	Gastropods	*Crepidula sp. fornicata*
September 1, 2021	Arousa. Bohido	Arousa	Bivalves	*Gari depressa*
September 1, 2021	Arousa. Bohido	Arousa	Bivalves	*Lutraria sp.*
September 1, 2021	Arousa. Bohido	Arousa	Polychaetes	*Sipunculus nudus*
September 1, 2021	Arousa. Bohido	Arousa	Crustaceans	*Atelecyclus undecimdentatus*
November 9, 2021	Rianxo	Arousa	Gastropods	*Crepidula sp.*
November 9, 2021	Rianxo	Arousa	Bivalves	*Aequipecten opercularis*
November 9, 2021	Rianxo	Arousa	Bivalves	*Pecten maximus*
November 9, 2021	Rianxo	Arousa	Bivalves	*Chlamys varia*
September 3, 2021	Vigo II.2	Vigo	Bivalves	*Ruditapes philippinarum*
September 7, 2021	Barqueiro. Pr. Salgueira	Barqueiro	Bivalves	*Mytilus galloprovincialis*
September 8, 2021	Barallobre. Rampa	Ferrol	Bivalves	*Mytilus galloprovincialis*
September 7, 2021	Miño. Lombo Espiñeira	Betanzos	Bivalves	*Ruditapes philippinarum*
September 7, 2021	Pasaxe. Sta Cristina	A Coruña	Bivalves	*Cerastoderma edule*
September 7, 2021	Camariñas. Rio da Ponte	Camariñas	Bivalves	*Ruditapes decussatus*
September 8, 2021	Corcubión. Pr.Cee	Corcubión	Bivalves	*Cerastoderma edule*
September 7, 2021	Muros I. Abelleira	Muros-Noia	Bivalves	*Cerastoderma edule*

September 7, 2021	Muros III. Testal	Muros-Noia	Bivalves	*Cerastoderma edule*
September 7, 2021	Pont V. Placeres	Pontevedra	Bivalves	*Ruditapes philippinarum*
September 15, 2021	Barqueiro. Pr. Salgueira	Barqueiro	Bivalves	*Mytilus galloprovincialis*
September 15, 2021	Barallobre. Rampa	Ferrol	Bivalves	*Mytilus galloprovincialis*
September 14, 2021	Miño. Lombo Espiñeira	Betanzos	Bivalves	*Ruditapes philippinarum*
September 14, 2021	Pasaxe. Sta Cristina	A Coruña	Bivalves	*Cerastoderma edule*
September 16, 2021	Camariñas. Ariño	Camariñas	Bivalves	*Ruditapes decussatus*
September 14, 2021	Corcubión. Pr.Cee	Corcubión	Bivalves	*Cerastoderma edule*
September 13, 2021	Muros III	Muros-Noia	Bivalves	*Cerastoderma edule*
September 14, 2021	Arousa I. Rons	Arousa	Bivalves	*Venerupis pullastra*
September 14, 2021	Pont V. Placeres	Pontevedra	Bivalves	*Ruditapes philippinarum*
September 16, 2021	Vigo I. Cies	Vigo	Bivalves	*Venerupis rhomboides*
September 14, 2021	Vigo II.2 Xunqueira	Vigo	Bivalves	*Ruditapes philippinarum*
September 21, 2021	Barqueiro. Pr. Salgueira	Barqueiro	Bivalves	*Mytilus galloprovincialis*
September 23, 2021	Barallobre. Rampa	Ferrol	Bivalves	*Mytilus galloprovincialis*
September 21, 2021	Miño. Lombo Espiñeira	Betanzos	Bivalves	*Ruditapes philippinarum*
September 21, 2021	Pasaxe. Sta Cristina	A Coruña	Bivalves	*Cerastoderma edule*
September 21, 2021	Camariñas. Enseada Vasa	Camariñas	Bivalves	*Ruditapes decussatus*
September 22, 2021	Corcubión. Pr.Cee	Corcubión	Bivalves	*Mytilus galloprovincialis*
September 21, 2021	Muros III	Muros-Noia	Bivalves	*Cerastoderma edule*
September 24, 2021	Arousa VII	Arousa	Bivalves	*Ruditapes philippinarum*
September 20, 2021	Pont V. Placeres	Pontevedra	Bivalves	*Ruditapes philippinarum*
September 22, 2021	Vigo I. Cies	Vigo	Bivalves	*Venerupis rhomboides*
September 20, 2021	Vigo II.2	Vigo	Bivalves	*Ruditapes philippinarum*
September 30, 2021	Barqueiro. Pr. Salgueira	Barqueiro	Bivalves	*Mytilus galloprovincialis*
September 28, 2021	Miño. Lombo Espiñeira	Betanzos	Bivalves	*Ruditapes philippinarum*
September 28, 2021	Pasaxe. Sta Cristina	A Coruña	Bivalves	*Cerastoderma edule*
September 29, 2021	Camariñas. Enseada Vasa	Camariñas	Bivalves	*Ruditapes decussatus*
September 29, 2021	Corcubión. Pr.Cee	Corcubión	Bivalves	*Mytilus galloprovincialis*

September 28, 2021	Muros I. Abelleira	Muros-Noia	Bivalves	*Cerastoderma edule*
September 27, 2021	Muros III. Testal	Muros-Noia	Bivalves	*Cerastoderma edule*
October 1, 2021	Arousa VII. Lombo das os	Arousa	Bivalves	*Ruditapes philippinarum*
September 28, 2021	Pont V. Placeres	Pontevedra	Bivalves	*Ruditapes philippinarum*
September 28, 2021	Vigo I. Cies	Vigo	Bivalves	*Venerupis rhomboides*
October 1, 2021	Vigo II.2 Xunqueira	Vigo	Bivalves	*Ruditapes philippinarum*
October 5, 2021	Barqueiro. Pr. Salgueira	Barqueiro	Bivalves	*Mytilus galloprovincialis*
October 5, 2021	Miño. Lombo Espiñeira	Betanzos	Bivalves	*Ruditapes philippinarum*
October 5, 2021	Pasaxe. Sta Cristina	A Coruña	Bivalves	*Cerastoderma edule*
October 5, 2021	Camariñas. Enseada Vasa	Camariñas	Bivalves	*Ruditapes decussatus*
October 5, 2021	Corcubión. Pr.Cee	Corcubión	Bivalves	*Cerastoderma edule*
October 4, 2021	Muros III. Testal	Muros-Noia	Bivalves	*Cerastoderma edule*
October 5, 2021	Pont V	Pontevedra	Bivalves	*Ruditapes philippinarum*
October 14, 2021	Barqueiro. Pr. Salgueira	Barqueiro	Bivalves	*Mytilus galloprovincialis*
October 14, 2021	Miño. Lombo Espiñeira	Betanzos	Bivalves	*Ruditapes philippinarum*
October 14, 2021	Pasaxe. Sta Cristina	A Coruña	Bivalves	*Cerastoderma edule*
October 14, 2021	Camariñas. Enseada Vasa	Camariñas	Bivalves	*Mytilus galloprovincialis*
October 13, 2021	Corcubión. Pr.Cee	Corcubión	Bivalves	*Cerastoderma edule*
October 14, 2021	Pont V. Placeres	Pontevedra	Bivalves	*Ruditapes philippinarum*
October 19, 2021	Barqueiro. Pr. Salgueira	Barqueiro	Bivalves	*Mytilus galloprovincialis*
October 19, 2021	Barallobre. Rampa	Ferrol	Bivalves	*Mytilus galloprovincialis*
October 19, 2021	Miño. Lombo Espiñeira	Betanzos	Bivalves	*Ruditapes philippinarum*
October 19, 2021	Pasaxe. Sta Cristina	A Coruña	Bivalves	*Cerastoderma edule*
October 19, 2021	Camariñas. Rio da Ponte	Camariñas	Bivalves	*Ruditapes philippinarum*
October 19, 2021	Corcubión. Pr.Cee	Corcubión	Bivalves	*Mytilus galloprovincialis*
October 19, 2021	Muros III. Testal	Muros-Noia	Bivalves	*Cerastoderma edule*
October 21, 2021	Pont V. Placeres	Pontevedra	Bivalves	*Ruditapes philippinarum*
November 30, 2021	Carnota. Lira	Corcubión	Echinoderms	*Paracentrotus sp.*
January 20, 2022	Vilanova de Arousa. As Sinas	Arousa	Cnidarias	*Actinia sp.*

January 20, 2022	Vilanova de Arousa. As Sinas	Arousa	Cnidarias	*Actinia sp.*
January 20, 2022	Vilanova de Arousa. As Sinas	Arousa	Gastropods	*Monodonta sp.*
January 20, 2022	Vilanova de Arousa. As Sinas	Arousa	Gastropods	*Nucella sp.*
January 20, 2022	Vilanova de Arousa. As Sinas	Arousa	Gastropods	*Patella sp.*
January 20, 2022	Vilanova de Arousa. As Sinas	Arousa	Crustaceans	*Balanus sp.*
January 20, 2022	Vilanova de Arousa. As Sinas	Arousa	Bivalves	*Mytilus galloprovincialis*
January 21, 2022	Vilanova de Arousa. Corón	Arousa	Crustaceans	*Balanus sp.*
January 21, 2022	Vilanova de Arousa. Corón	Arousa	Gastropods	*Patella sp.*
January 21, 2022	Vilanova de Arousa. Corón	Arousa	Cnidarias	*Actinia sp.*
January 21, 2022	Vilanova de Arousa. Corón	Arousa	Bivalves	*Mytilus galloprovincialis*
January 21, 2022	Vilanova de Arousa. Corón	Arousa	Gastropods	*Nucella sp.*
January 21, 2022	Vilanova de Arousa. Corón	Arousa	Gastropods	*Monodonta sp.*
January 21, 2022	Vilanova de Arousa. Corón	Arousa	Gastropods	*Littorina sp.*
October 22, 2021	Pont II. San Cibrao	Pontevedra	Bivalves	*Ruditapes philippinarum*
October 27, 2021	Barqueiro. Pr. Salgueira	Barqueiro	Bivalves	*Mytilus galloprovincialis*
October 26, 2021	Barallobre. Rampa	Ferrol	Bivalves	*Mytilus galloprovincialis*
October 26, 2021	Miño. Lombo Espiñeira	Betanzos	Bivalves	*Cerastoderma edule*
October 26, 2021	Pasaxe. Sta Cristina	A Coruña	Bivalves	*Cerastoderma edule*
October 26, 2021	Camariñas. Enseada Vasa	Camariñas	Bivalves	*Mytilus galloprovincialis*
October 26, 2021	Corcubión. Pr.Cee	Corcubión	Bivalves	*Mytilus galloprovincialis*
October 27, 2021	Pont V	Pontevedra	Bivalves	*Ruditapes philippinarum*
October 29, 2021	Vigo I. Limens	Vigo	Bivalves	*Ruditapes philippinarum*
October 27, 2021	Vigo II.2 Xunqueira	Vigo	Bivalves	*Ruditapes philippinarum*
November 3, 2021	Barqueiro. Pr. Salgueira	Barqueiro	Bivalves	*Mytilus galloprovincialis*
November 4, 2021	Barallobre. Rampa	Ferrol	Bivalves	*Mytilus galloprovincialis*
November 3, 2021	Miño. Lombo Espiñeira	Betanzos	Bivalves	*Cerastoderma edule*
November 3, 2021	Pasaxe. Sta Cristina	A Coruña	Bivalves	*Cerastoderma edule*
November 3, 2021	Camariñas. Enseada Vasa	Camariñas	Bivalves	*Mytilus galloprovincialis*
November 4, 2021	Corcubión. Pr.Cee	Corcubión	Bivalves	*Mytilus galloprovincialis*

November 3, 2021	Muros III. Testal	Muros-Noia	Bivalves	*Cerastoderma edule*
November 3, 2021	Vigo I. Liméns	Vigo	Bivalves	*Venerupis rhomboides*
November 3, 2021	Vigo II.2. Tirán	Vigo	Bivalves	*Venerupis rhomboides*
November 9, 2021	Barqueiro. Pr. Salgueira	Barqueiro	Bivalves	*Mytilus galloprovincialis*
November 9, 2021	Barallobre. Rampa	Ferrol	Bivalves	*Ruditapes philippinarum*
November 9, 2021	Miño. Lombo Espiñeira	Betanzos	Bivalves	*Cerastoderma edule*
November 9, 2021	Pasaxe. Sta Cristina	A Coruña	Bivalves	*Cerastoderma edule*
November 9, 2021	Camariñas. Río da Ponte	Camariñas	Bivalves	*Mytilus galloprovincialis*
November 11, 2021	Corcubión. Pr.Cee	Corcubión	Bivalves	*Mytilus galloprovincialis*
November 9, 2021	Pont V. Placeres	Pontevedra	Bivalves	*Ruditapes philippinarum*
November 10, 2021	Vigo I	Vigo	Bivalves	*Venerupis rhomboides*
November 11, 2021	Vigo II.2 Tirán	Vigo	Bivalves	*Venerupis rhomboides*
November 17, 2021	Barqueiro. Pr. Salgueira	Barqueiro	Bivalves	*Mytilus galloprovincialis*
November 16, 2021	Barallobre. Rampa	Ferrol	Bivalves	*Mytilus galloprovincialis*
November 17, 2021	Miño. Muro Petra Sabio	Betanzos	Bivalves	*Mytilus galloprovincialis*
November 16, 2021	Pasaxe. Sta Cristina	A Coruña	Bivalves	*Cerastoderma edule*
November 16, 2021	Camariñas. Río da ponte	Camariñas	Bivalves	*Mytilus galloprovincialis*
November 16, 2021	Corcubión. Pr.Cee	Corcubión	Bivalves	*Mytilus galloprovincialis*
November 24, 2021	Barqueiro. Pr. Salgueira	Barqueiro	Bivalves	*Mytilus galloprovincialis*
November 23, 2021	Barallobre. Rampa	Ferrol	Bivalves	*Mytilus galloprovincialis*
November 23, 2021	Miño. Muro Petra Sabio	Betanzos	Bivalves	*Mytilus galloprovincialis*
November 23, 2021	Pasaxe. Sta Cristina	A Coruña	Bivalves	*Cerastoderma edule*
November 23, 2021	Camariñas. Enseada Vasa	Camariñas	Bivalves	*Mytilus galloprovincialis*
November 23, 2021	Corcubión. Pr.Cee	Corcubión	Bivalves	*Mytilus galloprovincialis*
November 26, 2021	Vigo I	Vigo	Bivalves	*Venerupis rhomboides*
November 30, 2021	Barqueiro. Pr. Salgueira	Barqueiro	Bivalves	*Mytilus galloprovincialis*
November 30, 2021	Barallobre. Rampa	Ferrol	Bivalves	*Mytilus galloprovincialis*
November 30, 2021	Miño. Muro Petra Sabio	Betanzos	Bivalves	*Mytilus galloprovincialis*
November 30, 2021	Pasaxe. Sta Cristina	A Coruña	Bivalves	*Cerastoderma edule*

December 2, 2021	Camariñas. Enseada Vasa	Camariñas	Bivalves	*Mytilus galloprovincialis*
November 30, 2021	Corcubión. Pr.Cee	Corcubión	Bivalves	*Mytilus galloprovincialis*
December 9, 2021	Barqueiro. Pr. Salgueira	Barqueiro	Bivalves	*Mytilus galloprovincialis*
December 9, 2021	Barallobre. Rampa	Ferrol	Bivalves	*Mytilus galloprovincialis*
December 9, 2021	Miño. Muro Petra Sabio	Betanzos	Bivalves	*Mytilus galloprovincialis*
December 9, 2021	Pasaxe. Sta Cristina	A Coruña	Bivalves	*Cerastoderma edule*
December 9, 2021	Camariñas. Enseada Vasa	Camariñas	Bivalves	*Mytilus galloprovincialis*
December 10, 2021	Corcubión. Pr.Cee	Corcubión	Bivalves	*Mytilus galloprovincialis*
December 15, 2021	Barqueiro. Pr. Salgueira	Barqueiro	Bivalves	*Mytilus galloprovincialis*
December 14, 2021	Barallobre. Rampa	Ferrol	Bivalves	*Mytilus galloprovincialis*
December 14, 2021	Miño. Muro Petra Sabio	Betanzos	Bivalves	*Mytilus galloprovincialis*
December 14, 2021	Pasaxe. Sta Cristina	A Coruña	Bivalves	*Cerastoderma edule*
December 16, 2021	Camariñas. Enseada Vasa	Camariñas	Bivalves	*Mytilus galloprovincialis*
December 15, 2021	Corcubión. Pr.Cee	Corcubión	Bivalves	*Mytilus galloprovincialis*
February 4, 2022	Carnota. Lira	Corcubión	Echinoderms	*Paracentrotus sp.*
December 21, 2021	Barqueiro. Pr. Salgueira	Barqueiro	Bivalves	*Mytilus galloprovincialis*
December 21, 2021	Barallobre. Rampa	Ferrol	Bivalves	*Mytilus galloprovincialis*
December 21, 2021	Miño. Muro Petra Sabio	Betanzos	Bivalves	*Mytilus galloprovincialis*
December 22, 2021	Pasaxe. Sta Cristina	A Coruña	Bivalves	*Cerastoderma edule*
December 21, 2021	Camariñas. Enseada Vasa	Camariñas	Bivalves	*Mytilus galloprovincialis*
December 23, 2021	Corcubión. Pr.Cee	Corcubión	Bivalves	*Mytilus galloprovincialis*
December 29, 2021	Barqueiro. Pr. Salgueira	Barqueiro	Bivalves	*Mytilus galloprovincialis*
December 28, 2021	Barallobre. Rampa	Ferrol	Bivalves	*Mytilus galloprovincialis*
December 28, 2021	Miño. Muro Petra Sabio	Betanzos	Bivalves	*Mytilus galloprovincialis*
December 28, 2021	Pasaxe. Sta Cristina	A Coruña	Bivalves	*Cerastoderma edule*
December 28, 2021	Camariñas. Río da Ponte	Camariñas	Bivalves	*Mytilus galloprovincialis*
December 28, 2021	Corcubión. Pr.Cee	Corcubión	Bivalves	*Mytilus galloprovincialis*
January 4, 2022	Barqueiro. Pr. Salgueira	Barqueiro	Bivalves	*Mytilus galloprovincialis*
January 4, 2022	Barallobre. Pantalán	Ferrol	Bivalves	*Mytilus galloprovincialis*

January 4, 2022	Miño. Muro Petra Sabio	Betanzos	Bivalves	*Mytilus galloprovincialis*
January 4, 2022	Pasaxe. Sta Cristina	A Coruña	Bivalves	*Cerastoderma edule*
January 4, 2022	Camariñas. Enseada Vasa	Camariñas	Bivalves	*Mytilus galloprovincialis*
January 5, 2022	Corcubión. Pr.Cee	Corcubión	Bivalves	*Mytilus galloprovincialis*
January 12, 2022	Barqueiro. Pr. Salgueira	Barqueiro	Bivalves	*Mytilus galloprovincialis*
January 11, 2022	Barallobre. Rampa	Ferrol	Bivalves	*Mytilus galloprovincialis*
January 11, 2022	Miño. Muro Petra Sabio	Betanzos	Bivalves	*Mytilus galloprovincialis*
January 11, 2022	Pasaxe. Sta Cristina	A Coruña	Bivalves	*Cerastoderma edule*
January 11, 2022	Camariñas. Enseada Vasa	Camariñas	Bivalves	*Mytilus galloprovincialis*
January 11, 2022	Corcubión. Pr.Cee	Corcubión	Bivalves	*Mytilus galloprovincialis*
January 20, 2022	Barqueiro. Pr. Salgueira	Barqueiro	Bivalves	*Mytilus galloprovincialis*
January 19, 2022	Barallobre. Rampa	Ferrol	Bivalves	*Mytilus galloprovincialis*
January 18, 2022	Miño. Muro Petra Sabio	Betanzos	Bivalves	*Mytilus galloprovincialis*
January 18, 2022	Pasaxe. Sta Cristina	A Coruña	Bivalves	*Mytilus galloprovincialis*
January 18, 2022	Camariñas. Enseada Vasa	Camariñas	Bivalves	*Mytilus galloprovincialis*
January 18, 2022	Corcubión. Pr.Cee	Corcubión	Bivalves	*Mytilus galloprovincialis*
January 27, 2022	Barqueiro. Pr. Salgueira	Barqueiro	Bivalves	*Mytilus galloprovincialis*
January 25, 2022	Barallobre. Rampa	Ferrol	Bivalves	*Mytilus galloprovincialis*
January 25, 2022	Miño. Muro Petra Sabio	Betanzos	Bivalves	*Mytilus galloprovincialis*
January 25, 2022	Pasaxe. Sta Cristina	A Coruña	Bivalves	*Cerastoderma edule*
January 25, 2022	Camariñas. Enseada Vasa	Camariñas	Bivalves	*Mytilus galloprovincialis*
January 26, 2022	Corcubión. Pr.Cee	Corcubión	Bivalves	*Mytilus galloprovincialis*
February 1, 2022	Barqueiro. Pr. Salgueira	Barqueiro	Bivalves	*Mytilus galloprovincialis*
February 1, 2022	Barallobre. Rampa	Ferrol	Bivalves	*Mytilus galloprovincialis*
February 1, 2022	Miño. Muro Petra Sabio	Betanzos	Bivalves	*Mytilus galloprovincialis*
February 1, 2022	Pasaxe. Sta Cristina	A Coruña	Bivalves	*Cerastoderma edule*
February 1, 2022	Camariñas. Enseada Vasa	Camariñas	Bivalves	*Mytilus galloprovincialis*
February 1, 2022	Corcubión. Pr.Cee	Corcubión	Bivalves	*Mytilus galloprovincialis*
February 9, 2022	Barqueiro. Pr. Salgueira	Barqueiro	Bivalves	*Mytilus galloprovincialis*

February 8, 2022	Barallobre. Rampa	Ferrol	Bivalves	*Mytilus galloprovincialis*
February 8, 2022	Miño. Muro Petra Sabio	Betanzos	Bivalves	*Mytilus galloprovincialis*
February 8, 2022	Pasaxe. Sta Cristina	A Coruña	Bivalves	*Cerastoderma edule*
February 8, 2022	Camariñas. Enseada Vasa	Camariñas	Bivalves	*Mytilus galloprovincialis*
February 8, 2022	Corcubión. Pr.Cee	Corcubión	Bivalves	*Mytilus galloprovincialis*
February 9, 2022	Muros III	Muros-Noia	Bivalves	*Cerastoderma edule*
February 15, 2022	Barqueiro. Pr. Salgueira	Barqueiro	Bivalves	*Mytilus galloprovincialis*
February 15, 2022	Barallobre. Rampa	Ferrol	Bivalves	*Mytilus galloprovincialis*
February 15, 2022	Miño. Muro Petra Sabio	Betanzos	Bivalves	*Mytilus galloprovincialis*
February 15, 2022	Pasaxe. Sta Cristina	A Coruña	Bivalves	*Cerastoderma edule*
February 15, 2022	Camariñas. Enseada Vasa	Camariñas	Bivalves	*Mytilus galloprovincialis*
February 15, 2022	Corcubión. Pr.Cee	Corcubión	Bivalves	*Mytilus galloprovincialis*
February 14, 2022	Muros III	Muros-Noia	Bivalves	*Cerastoderma edule*
February 15, 2022	Pont V. Placeres	Pontevedra	Bivalves	*Ruditapes philippinarum*
February 21, 2022	Arousa I.	Arousa	Bivalves	*Aequipecten opercularis*
March 2, 2022	Carnota. Lira	Corcubión	Echinoderms	*Paracentrotus sp.*
March 2, 2022	Pont V. Placeres	Pontevedra	Gastropods	*Littorina sp.*
March 2, 2022	Pont V. Placeres	Pontevedra	Bivalves	*Ostrea edulis*
March 2, 2022	Pont V. Placeres	Pontevedra	Gastropods	*Patella sp.*
March 2, 2022	Pont V. Placeres	Pontevedra	Bivalves	*Ruditapes philippinarum*
March 2, 2022	Pont V. Placeres	Pontevedra	Bivalves	*Mytilus galloprovincialis*
March 4, 2022	Vilanova de Arousa. Corón	Arousa	Bivalves	*Cerastoderma edule*
March 4, 2022	Vilanova de Arousa. Corón	Arousa	Gastropods	*n.i.*
March 4, 2022	Vilanova de Arousa. Corón	Arousa	Crustaceans	*Polybius sp.*
March 4, 2022	Vilanova de Arousa. Corón	Arousa	Bivalves	*Mytilus galloprovincialis*
March 4, 2022	Vilanova de Arousa. Corón	Arousa	Gastropods	*Littorina sp.*
March 4, 2022	Vilanova de Arousa. Corón	Arousa	Gastropods	*Nucella sp.*
March 4, 2022	Vilanova de Arousa. Corón	Arousa	Poriferous	*n.i.*
March 4, 2022	Vilanova de Arousa. Corón	Arousa	Gastropods	*Nucella sp.*

March 4, 2022	Vilanova de Arousa. Corón	Arousa	Gastropods	*Patella sp.*
March 4, 2022	Vilanova de Arousa. Corón	Arousa	Gastropods	*Nassarius sp.*
March 4, 2022	Vilanova de Arousa. Corón	Arousa	Bivalves	*Ruditapes philippinarum*
March 4, 2022	Vilanova de Arousa. Corón	Arousa	Gastropods	*Monodonta sp.*
March 4, 2022	Vilanova de Arousa. Corón	Arousa	Crustaceans	*Balanus sp.*
March 4, 2022	Vilanova de Arousa. Corón	Arousa	Cnidarias	*n.i.*
March 4, 2022	Porto do Son. Arnela	Muros-Noia	Bivalves	*Mytilus galloprovincialis*
March 4, 2022	Porto do Son. Arnela	Muros-Noia	Gastropods	*Patella sp.*
March 4, 2022	Porto do Son. Arnela	Muros-Noia	Crustaceans	*Balanus sp.*
March 4, 2022	Porto do Son. Arnela	Muros-Noia	Cnidarias	*n.i.*
March 4, 2022	Porto do Son. Arnela	Muros-Noia	Cnidarias	*n.i.*
March 4, 2022	Porto do Son. Arnela	Muros-Noia	Polychaetes	*n.i.*
March 4, 2022	Porto do Son. Arnela	Muros-Noia	Gastropods	*Monodonta sp.*
March 4, 2022	Porto do Son. Arnela	Muros-Noia	Gastropods	*Gibbula sp.*
March 4, 2022	Porto do Son. Arnela	Muros-Noia	Gastropods	*Nucella sp.*
April 8, 2022	Nigrán. Patos	Vigo	Gastropods	*Monodonta sp.*
April 8, 2022	Nigrán. Patos	Vigo	Gastropods	*Patella sp.*
April 8, 2022	Nigrán. Patos	Vigo	Gastropods	*n.i.*
April 6, 2022	Arousa. Bohido	Arousa	Bivalves	*Venus verrucosa*
April 6, 2022	Arousa. Bohido	Arousa	Bivalves	*Dosinia exoleta*
April 18, 2022	Porto do Son. Arnela	Muros-Noia	Gastropods	*Nucella sp.*
April 18, 2022	Porto do Son. Arnela	Muros-Noia	Gastropods	*Monodonta sp.*
April 18, 2022	Porto do Son. Arnela	Muros-Noia	Gastropods	*Gibbula sp.*
April 18, 2022	Porto do Son. Arnela	Muros-Noia	Echinoderms	*Asterina sp.*
April 18, 2022	Porto do Son. Arnela	Muros-Noia	Gastropods	*Patella sp.*
April 18, 2022	Porto do Son. Arnela	Muros-Noia	Echinoderms	*Paracentrotus sp.*
April 18, 2022	Porto do Son. Arnela	Muros-Noia	Cnidarias	*n.i.*
April 18, 2022	Porto do Son. Arnela	Muros-Noia	Bivalves	*Mytilus galloprovincialis*
April 18, 2022	Porto do Son. Arnela	Muros-Noia	Polychaetes	*n.i.*

April 19, 2022	Vilanova de Arousa. Corón	Arousa	Crustaceans	*Polybius sp.*
April 19, 2022	Vilanova de Arousa. Corón	Arousa	Bivalves	*Cerastoderma edule*
April 19, 2022	Vilanova de Arousa. Corón	Arousa	Bivalves	*Ruditapes philippinarum*
April 19, 2022	Vilanova de Arousa. Corón	Arousa	Gastropods	*Littorina sp.*
April 19, 2022	Vilanova de Arousa. Corón	Arousa	Cnidarias	*n.i.*
April 19, 2022	Vilanova de Arousa. Corón	Arousa	Bivalves	*Mytilus galloprovincialis*
April 19, 2022	Vilanova de Arousa. Corón	Arousa	Gastropods	*Patella sp.*
April 19, 2022	Vilanova de Arousa. Corón	Arousa	Crustaceans	*Balanus sp.*
April 19, 2022	Vilanova de Arousa. Corón	Arousa	Gastropods	*Monodonta sp.*
April 19, 2022	Vilanova de Arousa. Corón	Arousa	Gastropods	*Nassarius sp.*
April 19, 2022	Vilanova de Arousa. Corón	Arousa	Gastropods	*Ocenebra sp.*
April 19, 2022	Vilanova de Arousa. Corón	Arousa	Gastropods	*Nucella sp.*
April 19, 2022	Carnota. Lira	Corcubión	Echinoderms	*Paracentrotus sp.*
April 19, 2022	Aldán. Vilariño	Pontevedra	Gastropods	*Crepidula sp.*
April 19, 2022	Aldán. Vilariño	Pontevedra	Gastropods	*Monodonta sp.*
April 19, 2022	Aldán. Vilariño	Pontevedra	Gastropods	*Nucella sp.*
April 19, 2022	Aldán. Vilariño	Pontevedra	Crustaceans	*Polybius sp.*
April 19, 2022	Aldán. Vilariño	Pontevedra	Cnidarias	*n.i.*
April 19, 2022	Aldán. Vilariño	Pontevedra	Cnidarias	*n.i.*
April 19, 2022	Aldán. Vilariño	Pontevedra	Gastropods	*Patella sp.*
April 19, 2022	Aldán. Vilariño	Pontevedra	Bivalves	*Mytilus galloprovincialis*
April 19, 2022	Bueu. Beluso	Pontevedra	Gastropods	*Patella sp.*
April 19, 2022	Bueu. Beluso	Pontevedra	Cnidarias	*n.i.*
April 19, 2022	Bueu. Beluso	Pontevedra	Bivalves	*Mytilus galloprovincialis*
April 19, 2022	Bueu. Beluso	Pontevedra	Gastropods	*Crepidula sp.*
April 19, 2022	Bueu. Beluso	Pontevedra	Gastropods	*Monodonta sp.*
April 21, 2022	Pont V. Placeres	Pontevedra	Gastropods	*Monodonta sp.*
April 21, 2022	Pont V. Placeres	Pontevedra	Gastropods	*Littorina sp.*
April 21, 2022	Pont V. Placeres	Pontevedra	Gastropods	*Patella sp.*

April 21, 2022	Pont V. Placeres	Pontevedra	Crustaceans	*Polybius sp.*
April 21, 2022	Pont V. Placeres	Pontevedra	Bivalves	*Mytilus galloprovincialis*
May 2, 2022	Carnota. Lira	Corcubión	Echinoderms	*Paracentrotus sp.*
May 2, 2022	Arousa. Bohido	Arousa	Bivalves	*Ruditapes philippinarum*
May 2, 2022	Carnota. Lira	Corcubión	Gastropods	*Patella ulyssiponensi*
May 2, 2022	Carnota. Lira	Corcubión	Gastropods	*Patella vulgata*
May 2, 2022	Carnota. Lira	Corcubión	Gastropods	*Haliotis tuberculata*
May 2, 2022	Carnota. Lira	Corcubión	Echinoderms	*Holothuria forskali*
May 2, 2022	Carnota. Lira	Corcubión	Echinoderms	*Marthasterias glacialis*
February 23, 2022	Barqueiro. Pr. Salgueira	Barqueiro	Bivalves	*Mytilus galloprovincialis*
February 23, 2022	Barallobre. Rampa	Ferrol	Bivalves	*Mytilus galloprovincialis*
February 22, 2022	Miño. Muro Petra Sabio	Betanzos	Bivalves	*Mytilus galloprovincialis*
February 22, 2022	Pasaxe. Sta Cristina	A Coruña	Bivalves	*Cerastoderma edule*
February 22, 2022	Camariñas. Enseada Vasa	Camariñas	Bivalves	*Mytilus galloprovincialis*
February 22, 2022	Corcubión. Pr.Cee	Corcubión	Bivalves	*Mytilus galloprovincialis*
February 22, 2022	Muros III. Testal	Muros-Noia	Bivalves	*Cerastoderma edule*
March 1, 2022	Barqueiro. Pr. Salgueira	Barqueiro	Bivalves	*Mytilus galloprovincialis*
March 2, 2022	Barallobre. Rampa	Ferrol	Bivalves	*Mytilus galloprovincialis*
March 1, 2022	Miño. Muro Petra Sabio	Betanzos	Bivalves	*Mytilus galloprovincialis*
March 1, 2022	Pasaxe. Sta Cristina	A Coruña	Bivalves	*Cerastoderma edule*
March 1, 2022	Camariñas. Enseada Vasa	Camariñas	Bivalves	*Mytilus galloprovincialis*
March 2, 2022	Corcubión. Pr.Cee	Corcubión	Bivalves	*Cerastoderma edule*
March 8, 2022	Barqueiro. Pr. Salgueira	Barqueiro	Bivalves	*Mytilus galloprovincialis*
March 9, 2022	Barallobre. Rampa	Ferrol	Bivalves	*Mytilus galloprovincialis*
March 8, 2022	Miño. Muro Petra Sabio	Betanzos	Bivalves	*Mytilus galloprovincialis*
March 8, 2022	Pasaxe. Sta Cristina	A Coruña	Bivalves	*Cerastoderma edule*
March 8, 2022	Camariñas. Enseada Vasa	Camariñas	Bivalves	*Mytilus galloprovincialis*
March 8, 2022	Corcubión. Pr.Cee	Corcubión	Bivalves	*Mytilus galloprovincialis*
March 15, 2022	Barallobre. Rampa	Ferrol	Bivalves	*Mytilus galloprovincialis*

March 17, 2022	Miño. Muro Petra Sabio	Betanzos	Bivalves	*Mytilus galloprovincialis*
March 17, 2022	Camariñas. Enseada Vasa	Camariñas	Bivalves	*Mytilus galloprovincialis*
March 24, 2022	Barqueiro. Pr. Salgueira	Barqueiro	Bivalves	*Mytilus galloprovincialis*
March 25, 2022	Barallobre. Rampa	Ferrol	Bivalves	*Mytilus galloprovincialis*
March 22, 2022	Miño. Muro Petra Sabio	Betanzos	Bivalves	*Mytilus galloprovincialis*
March 22, 2022	Camariñas. Enseada Vasa	Camariñas	Bivalves	*Mytilus galloprovincialis*
March 23, 2022	Corcubión. Pr.Cee	Corcubión	Bivalves	*Mytilus galloprovincialis*
March 30, 2022	Barqueiro. Pr. Salgueira	Barqueiro	Bivalves	*Mytilus galloprovincialis*
March 29, 2022	Barallobre. Rampa	Ferrol	Bivalves	*Mytilus galloprovincialis*
March 29, 2022	Miño. Muro Petra Sabio	Betanzos	Bivalves	*Mytilus galloprovincialis*
March 29, 2022	Pasaxe. Sta Cristina	A Coruña	Bivalves	*Cerastoderma edule*
March 29, 2022	Camariñas. Enseada Vasa	Camariñas	Bivalves	*Mytilus galloprovincialis*
March 31, 2022	Corcubión. Pr.Cee	Corcubión	Bivalves	*Mytilus galloprovincialis*
March 30, 2022	Muros III	Muros-Noia	Bivalves	*Cerastoderma edule*
April 5, 2022	Barqueiro. Pr. Salgueira	Barqueiro	Bivalves	*Mytilus galloprovincialis*
April 5, 2022	Barallobre. Rampa	Ferrol	Bivalves	*Mytilus galloprovincialis*
April 5, 2022	Miño. Muro Petra Sabio	Betanzos	Bivalves	*Mytilus galloprovincialis*
April 5, 2022	Pasaxe. Sta Cristina	A Coruña	Bivalves	*Cerastoderma edule*
April 5, 2022	Camariñas. Enseada Vasa	Camariñas	Bivalves	*Mytilus galloprovincialis*
April 5, 2022	Muros I	Muros-Noia	Bivalves	*Ensis ensis*
April 4, 2022	Muros III. Testal	Muros-Noia	Bivalves	*Cerastoderma edule*
April 5, 2022	Pont II	Pontevedra	Bivalves	*Ensis ensis*
April 6, 2022	Camariñas. Enseada Vasa	Camariñas	Bivalves	*Ruditapes decussatus*
April 6, 2022	Corcubión. Pr.Cee	Corcubión	Bivalves	*Mytilus galloprovincialis*
April 6, 2022	Muros I	Muros-Noia	Bivalves	*Cerastoderma edule*
April 6, 2022	Muros III	Muros-Noia	Bivalves	*Cerastoderma edule*
April 12, 2022	Barallobre. Rampa	Ferrol	Bivalves	*Cerastoderma edule*
April 12, 2022	Miño. Lombo Espiñeira	Betanzos	Bivalves	*Cerastoderma edule*
April 12, 2022	Pasaxe. Sta Cristina	A Coruña	Bivalves	*Cerastoderma edule*

April 12, 2022	Muros I. Cabeiro	Muros-Noia	Bivalves	*Ensis ensis*
April 11, 2022	Muros III. Testal	Muros-Noia	Bivalves	*Cerastoderma edule*
April 13, 2022	Barqueiro. Pr. Salgueira	Barqueiro	Bivalves	*Cerastoderma edule*
April 13, 2022	Camariñas. Enseada Vasa	Camariñas	Bivalves	*Ruditapes decussatus*
April 13, 2022	Corcubión. Pr.Cee	Corcubión	Bivalves	*Cerastoderma edule*
April 13, 2022	Muros I. Abelleira	Muros-Noia	Bivalves	*Cerastoderma edule*
April 19, 2022	Barqueiro. Pr. Salgueira	Barqueiro	Bivalves	*Mytilus galloprovincialis*
April 19, 2022	Barallobre. Rampa	Ferrol	Bivalves	*Mytilus galloprovincialis*
April 19, 2022	Miño. Muro Petra Sabio	Betanzos	Bivalves	*Mytilus galloprovincialis*
April 20, 2022	Pasaxe. Sta Cristina	A Coruña	Bivalves	*Cerastoderma edule*
April 19, 2022	Camariñas. Enseada Vasa	Camariñas	Bivalves	*Ruditapes decussatus*
April 20, 2022	Corcubión. Pr.Cee	Corcubión	Bivalves	*Mytilus galloprovincialis*
April 19, 2022	Muros I	Muros-Noia	Bivalves	*Ensis ensis*
April 18, 2022	Muros III. Testal	Muros-Noia	Bivalves	*Cerastoderma edule*
April 22, 2022	Arousa I.	Arousa	Bivalves	*Venerupis rhomboides*
April 21, 2022	Pont II	Pontevedra	Bivalves	*Ensis ensis*
April 22, 2022	Pont V	Pontevedra	Bivalves	*Ruditapes philippinarum*
April 21, 2022	Vigo II.2	Vigo	Bivalves	*Ruditapes philippinarum*
April 26, 2022	Barqueiro. Pr. Salgueira	Barqueiro	Bivalves	*Mytilus galloprovincialis*
April 26, 2022	Barallobre. Rampa	Ferrol	Bivalves	*Mytilus galloprovincialis*
April 26, 2022	Miño. Muro Petra Sabio	Betanzos	Bivalves	*Mytilus galloprovincialis*
April 27, 2022	Pasaxe. Sta Cristina	A Coruña	Bivalves	*Cerastoderma edule*
April 26, 2022	Camariñas. Enseada Vasa	Camariñas	Bivalves	*Mytilus galloprovincialis*
April 26, 2022	Corcubión. Pr.Cee	Corcubión	Bivalves	*Mytilus galloprovincialis*
April 27, 2022	Muros I	Muros-Noia	Bivalves	*Cerastoderma edule*
April 29, 2022	Arousa I.	Arousa	Bivalves	*Ruditapes philippinarum*
April 28, 2022	Pont II	Pontevedra	Bivalves	*Ensis ensis*
April 28, 2022	Pont V	Pontevedra	Bivalves	*Ruditapes philippinarum*
April 29, 2022	Barallobre. Rampa	Ferrol	Bivalves	*Ruditapes philippinarum*

May 3, 2022	Barqueiro. Pr. Salgueira	Barqueiro	Bivalves	*Magallana gigas*
May 3, 2022	Barallobre. Maniños	Ferrol	Bivalves	*Ruditapes philippinarum*
May 3, 2022	Miño. Lombo Espiñeira	Betanzos	Bivalves	*Ruditapes philippinarum*
May 4, 2022	Pasaxe. Sta Cristina	A Coruña	Bivalves	*Cerastoderma edule*
May 3, 2022	Camariñas. Enseada Vasa	Camariñas	Bivalves	*Ruditapes decussatus*
May 4, 2022	Corcubión. Pr.Cee	Corcubión	Bivalves	*Mytilus galloprovincialis*
May 3, 2022	Muros I	Muros-Noia	Bivalves	*Cerastoderma edule*
May 4, 2022	Pont V	Pontevedra	Bivalves	*Ensis ensis*
May 5, 2022	Pont V	Pontevedra	Bivalves	*Ruditapes philippinarum*
May 11, 2022	Barqueiro. Pr. Salgueira	Barqueiro	Bivalves	*Magallana gigas*
May 10, 2022	Barallobre. Maniños	Ferrol	Bivalves	*Cerastoderma edule*
May 11, 2022	Miño. Lombo Espiñeira	Betanzos	Bivalves	*Ruditapes philippinarum*
May 12, 2022	Pasaxe. Sta Cristina	A Coruña	Bivalves	*Cerastoderma edule*
May 11, 2022	Camariñas. Enseada Vasa	Camariñas	Bivalves	*Ruditapes decussatus*
May 10, 2022	Corcubión. Pr.Cee	Corcubión	Bivalves	*Cerastoderma edule*
May 12, 2022	Pont V. Placeres	Pontevedra	Bivalves	*Venerupis pullastra*
May 12, 2022	Vigo II.2 Xunqueira	Vigo	Gastropods	*Haliotis sp.*
May 26, 2022	*Carnota. Lira*	Corcubión	Echinoderms	*Paracentrotus sp.*
May 24, 2022	Arousa. Bohido	Arousa	Echinoderms	*Asterina gibbosa*
May 24, 2022	Arousa. Bohido	Arousa	Echinoderms	*Paracentrotus lividus*
May 24, 2022	Arousa. Bohido	Arousa	Cnidarias	*Calliactis parasitica*
May 24, 2022	Arousa. Bohido	Arousa	Crustaceans	*Nécora puber*
May 24, 2022	Arousa. Bohido	Arousa	Crustaceans	*Liocarcinus corrugatus*
May 24, 2022	Arousa. Bohido	Arousa	Crustaceans	*Liocarcinus arcuatus*
May 24, 2022	Arousa. Bohido	Arousa	Gastropods	*Nassarius reticulatus*
May 24, 2022	Arousa. Bohido	Arousa	Crustaceans	*Atelecyclus undecimdentatus*
June 14, 2022	Porto do Son. Arnela	Muros-Noia	Polychaetes	*n.i.*
June 14, 2022	Porto do Son. Arnela	Muros-Noia	Gastropods	*Shiponaria pectinata*
June 14, 2022	Porto do Son. Arnela	Muros-Noia	Bivalves	*Mytilus galloprovincialis*

June 14, 2022	Porto do Son. Arnela	Muros-Noia	Crustaceans	*Balanus sp.*
June 14, 2022	Porto do Son. Arnela	Muros-Noia	Echinoderms	*Asterina sp.*
June 14, 2022	Porto do Son. Arnela	Muros-Noia	Cnidarias	*n.i.*
June 14, 2022	Porto do Son. Arnela	Muros-Noia	Gastropods	*Nucella sp.*
June 14, 2022	Porto do Son. Arnela	Muros-Noia	Gastropods	*Patella sp.*
June 14, 2022	Porto do Son. Arnela	Muros-Noia	Gastropods	*Monodonta sp.*
June 14, 2022	Vilanova de Arousa. Corón	Arousa	Cnidarias	*n.i.*
June 14, 2022	Vilanova de Arousa. Corón	Arousa	Gastropods	*Nassarius sp.*
June 14, 2022	Vilanova de Arousa. Corón	Arousa	Gastropods	*Monodonta sp.*
June 14, 2022	Vilanova de Arousa. Corón	Arousa	Gastropods	*Gibbula sp.*
June 14, 2022	Vilanova de Arousa. Corón	Arousa	Crustaceans	*Polybius sp.*
June 14, 2022	Vilanova de Arousa. Corón	Arousa	Bivalves	*Ostrea edulis*
June 14, 2022	Vilanova de Arousa. Corón	Arousa	Crustaceans	*Balanus sp.*
June 14, 2022	Vilanova de Arousa. Corón	Arousa	Gastropods	*Patella sp.*
June 14, 2022	Vilanova de Arousa. Corón	Arousa	Bivalves	*Mytilus galloprovincialis*
June 14, 2022	Vilanova de Arousa. Corón	Arousa	Gastropods	*Nucella sp.*
June 14, 2022	Vilanova de Arousa. Corón	Arousa	Gastropods	*Littorina sp.*
June 14, 2022	Vilanova de Arousa. Corón	Arousa	Gastropods	*Doris verrucosa*
June 14, 2022	Bueu. Beluso	Pontevedra	Gastropods	*Patella sp.*
June 14, 2022	Bueu. Beluso	Pontevedra	Bivalves	*Mytilus galloprovincialis*
June 14, 2022	Bueu. Beluso	Pontevedra	Gastropods	*Monodonta sp.*
June 14, 2022	Bueu. Beluso	Pontevedra	Cnidarias	*n.i.*
June 14, 2022	Aldán. Vilariño	Pontevedra	Gastropods	*Littorina sp.*
June 14, 2022	Aldán. Vilariño	Pontevedra	Gastropods	*Patella sp.*
June 14, 2022	Aldán. Vilariño	Pontevedra	Gastropods	*Monodonta sp.*
June 14, 2022	Aldán. Vilariño	Pontevedra	Crustaceans	*Polybius sp.*
June 14, 2022	Aldán. Vilariño	Pontevedra	Cnidarias	*n.i.*
June 14, 2022	Aldán. Vilariño	Pontevedra	Bivalves	*Mytilus galloprovincialis*
June 14, 2022	Aldán. Vilariño	Pontevedra	Cnidarias	*n.i.*

June 17, 2022	Ramallosa. Esteiro Foz	Vigo	Gastropods	*Patella sp.*
June 17, 2022	Ramallosa. Esteiro Foz	Vigo	Bivalves	*Mytilus galloprovincialis*
June 17, 2022	Ramallosa. Esteiro Foz	Vigo	Bivalves	*Cerastoderma edule*
June 17, 2022	Ramallosa. Esteiro Foz	Vigo	Bivalves	*Ruditapes decussatus*
June 17, 2022	Ramallosa. Esteiro Foz	Vigo	Gastropods	*Littorina sp.*
June 17, 2022	Ramallosa. Esteiro Foz	Vigo	Crustaceans	*Carcinus maenas*
June 21, 2022	Pont V. Placeres	Pontevedra	Gastropods	*Littorina sp.*
June 21, 2022	Pont V. Placeres	Pontevedra	Bivalves	*Mytilus galloprovincialis*
June 21, 2022	Pont V. Placeres	Pontevedra	Gastropods	*Gibbula sp.*
June 21, 2022	Pont V. Placeres	Pontevedra	Crustaceans	*Polybius sp.*
June 21, 2022	Pont V. Placeres	Pontevedra	Gastropods	*Patella sp.*
June 21, 2022	Pont V. Placeres	Pontevedra	Gastropods	*Monodonta sp.*
July 19, 2022	Camariñas. Enseada Vasa	Camariñas	Gastropods	*Nassarius sp.*
July 19, 2022	Camariñas. Enseada Vasa	Camariñas	Gastropods	*Patella sp.*
July 19, 2022	Camariñas. Enseada Vasa	Camariñas	Bivalves	*Magallana gigas*
July 19, 2022	Camariñas. Enseada Vasa	Camariñas	Bivalves	*Mytilus galloprovincialis*
July 19, 2022	Camariñas. Enseada Vasa	Camariñas	Cnidarias	*n.i.*
July 19, 2022	Camariñas. Paxariñas	Camariñas	Bivalves	*Mytilus galloprovincialis*
July 19, 2022	Camariñas. Paxariñas	Camariñas	Crustaceans	*Polybius sp.*
July 19, 2022	Camariñas. Paxariñas	Camariñas	Gastropods	*Littorina sp.*
May 18, 2022	Barqueiro. Pr. Salgueira	Barqueiro	Bivalves	*Magallana gigas*
May 18, 2022	Barallobre. Maniños	Ferrol	Bivalves	*Cerastoderma edule*
May 18, 2022	Miño. Lombo Espiñeira	Betanzos	Bivalves	*Ruditapes philippinarum*
May 18, 2022	Pasaxe. Sta Cristina	A Coruña	Bivalves	*Cerastoderma edule*
May 18, 2022	Camariñas. Enseada Vasa	Camariñas	Bivalves	*Ruditapes decussatus*
May 19, 2022	Corcubión. Pr.Cee	Corcubión	Bivalves	*Cerastoderma edule*
May 25, 2022	Barqueiro. Pr. Salgueira	Barqueiro	Bivalves	*Magallana gigas*
May 26, 2022	Barallobre. Maniños	Ferrol	Bivalves	*Cerastoderma edule*
May 24, 2022	Miño. Lombo Espiñeira	Betanzos	Bivalves	*Ruditapes philippinarum*

May 24, 2022	Pasaxe. Sta Cristina	A Coruña	Bivalves	*Cerastoderma edule*
May 25, 2022	Camariñas. Enseada Vasa	Camariñas	Bivalves	*Ruditapes philippinarum*
May 25, 2022	Corcubión. Pr.Cee	Corcubión	Bivalves	*Cerastoderma edule*
May 25, 2022	Vigo I	Vigo	Bivalves	*Aequipecten opercularis*
May 24, 2022	Vigo II	Vigo	Gastropods	*Haliotis sp.*
June 1, 2022	Barqueiro. Pr. Salgueira	Barqueiro	Bivalves	*Magallana gigas*
June 1, 2022	Barallobre. Maniños	Ferrol	Bivalves	*Cerastoderma edule*
May 31, 2022	Miño. Lombo Espiñeira	Betanzos	Bivalves	*Ruditapes philippinarum*
June 1, 2022	Pasaxe. Sta Cristina	A Coruña	Bivalves	*Cerastoderma edule*
May 31, 2022	Camariñas. Enseada Vasa	Camariñas	Bivalves	*Ruditapes philippinarum*
June 1, 2022	Corcubión. Pr.Cee	Corcubión	Bivalves	*Mytilus galloprovincialis*
June 9, 2022	Barqueiro. Pr. Salgueira	Barqueiro	Bivalves	*Mytilus galloprovincialis*
June 8, 2022	Barallobre. Rampa	Ferrol	Bivalves	*Mytilus galloprovincialis*
June 7, 2022	Miño. Lombo Espiñeira	Betanzos	Bivalves	*Ruditapes philippinarum*
June 7, 2022	Pasaxe. Sta Cristina	A Coruña	Bivalves	*Cerastoderma edule*
June 7, 2022	Camariñas. Enseada Vasa	Camariñas	Bivalves	*Ruditapes philippinarum*
June 7, 2022	Corcubión. Pr.Cee	Corcubión	Bivalves	*Mytilus galloprovincialis*
June 14, 2022	Barqueiro. Pr. Salgueira	Barqueiro	Bivalves	*Mytilus galloprovincialis*
June 14, 2022	Barallobre. Rampa	Ferrol	Bivalves	*Mytilus galloprovincialis*
June 14, 2022	Miño. Lombo Espiñeira	Betanzos	Bivalves	*Ruditapes philippinarum*
June 14, 2022	Pasaxe. Sta Cristina	A Coruña	Bivalves	*Cerastoderma edule*
June 14, 2022	Camariñas. Enseada Vasa	Camariñas	Bivalves	*Ruditapes philippinarum*
June 14, 2022	Corcubión. Pr.Cee	Corcubión	Bivalves	*Mytilus galloprovincialis*
June 14, 2022	Camariñas. Zona E	Camariñas	Bivalves	*Ensis ensis*
June 23, 2022	Barqueiro. Pr. Salgueira	Barqueiro	Bivalves	*Mytilus galloprovincialis*
June 21, 2022	Barallobre. Pantalán	Ferrol	Bivalves	*Mytilus galloprovincialis*
June 21, 2022	Miño. Lombo Espiñeira	Betanzos	Bivalves	*Ruditapes philippinarum*
June 22, 2022	Pasaxe. Sta Cristina	A Coruña	Bivalves	*Cerastoderma edule*
June 21, 2022	Camariñas. Paxariñas	Camariñas	Bivalves	*Ruditapes philippinarum*

June 21, 2022	Pont II. Menduiña	Pontevedra	Bivalves	*Ensis ensis*
June 21, 2022	Camariñas. Zona E	Camariñas	Bivalves	*Ensis ensis*
June 28, 2022	Barqueiro. Pr. Salgueira	Barqueiro	Bivalves	*Mytilus galloprovincialis*
June 29, 2022	Barallobre. Rampa	Ferrol	Bivalves	*Mytilus galloprovincialis*
June 28, 2022	Miño. Lombo Espiñeira	Betanzos	Bivalves	*Ruditapes philippinarum*
June 28, 2022	Camariñas. Enseada Vasa	Camariñas	Bivalves	*Ruditapes philippinarum*
June 29, 2022	Corcubión. Pr.Cee	Corcubión	Bivalves	*Mytilus galloprovincialis*
June 30, 2022	Muros I. Cabeiro	Muros-Noia	Bivalves	*Ensis ensis*
July 1, 2022	Muros I. Pr. da Virxen	Muros-Noia	Bivalves	*Cerastoderma edule*
June 30, 2022	Pont II. San Cibrao	Pontevedra	Bivalves	*Ruditapes philippinarum*
June 30, 2022	Vigo I. Subrido	Vigo	Gastropods	*Haliotis sp.*
July 6, 2022	Barqueiro. Pr. Salgueira	Barqueiro	Bivalves	*Mytilus galloprovincialis*
July 5, 2022	Barallobre. Rampa	Ferrol	Bivalves	*Mytilus galloprovincialis*
July 5, 2022	Miño. Lombo Espiñeira	Betanzos	Bivalves	*Ruditapes philippinarum*
July 6, 2022	Pasaxe. Sta Cristina	A Coruña	Bivalves	*Cerastoderma edule*
July 5, 2022	Camariñas. Enseada Vasa	Camariñas	Bivalves	*Ruditapes decussatus*
July 5, 2022	Muros I. Abelleira	Muros-Noia	Bivalves	*Cerastoderma edule*
July 5, 2022	Muros I. Esteiro	Muros-Noia	Bivalves	*Ensis ensis*
July 5, 2022	Pont II. Niño do corvo	Pontevedra	Bivalves	*Ensis ensis*
July 7, 2022	Pont V	Pontevedra	Bivalves	*Ruditapes philippinarum*
July 12, 2022	Barqueiro. San Fiz	Barqueiro	Bivalves	*Cerastoderma edule*
July 12, 2022	Barallobre. Rampa	Ferrol	Bivalves	*Mytilus galloprovincialis*
July 12, 2022	Miño. Lombo Espiñeira	Betanzos	Bivalves	*Ruditapes philippinarum*
July 12, 2022	Camariñas. Enseada Vasa	Camariñas	Bivalves	*Ruditapes philippinarum*
July 12, 2022	Muros I. Pr. da Virxen	Muros-Noia	Bivalves	*Cerastoderma edule*
July 12, 2022	Pont II. Niño do corvo	Pontevedra	Bivalves	*Ensis ensis*
July 14, 2022	Pont V. Placeres	Pontevedra	Bivalves	*Ruditapes philippinarum*
July 11, 2022	Vigo II.2 Xunqueira	Vigo	Bivalves	*Ruditapes philippinarum*
July 14, 2022	Vigo II.2 Toralla	Vigo	Bivalves	*Venerupis pullastra*

July 14, 2022	Vigo II.2 Con. Norte	Vigo	Bivalves	*Ruditapes philippinarum*
July 7, 2022	Pont II	Pontevedra	Bivalves	*Venerupis rhomboides*
July 29, 2022	Arousa. Bohido	Arousa	Echinoderms	*Asteria rubens*
July 29, 2022	Arousa. Bohido	Arousa	Crustaceans	*Liocarcinus arcuatus*
July 29, 2022	Arousa. Bohido	Arousa	Crustaceans	*Chaetopleura angulata*
July 29, 2022	Arousa. Bohido	Arousa	Crustaceans	*Carcinus maenas*
July 29, 2022	Arousa. Bohido	Arousa	Crustaceans	*Liocarcinus corrugatus*
July 29, 2022	Arousa. Bohido	Arousa	Gastropods	*Crepidula dilatata*
September 10, 2022	Nerga. Os Castros	Vigo	Echinoderms	*Paracentrotus sp.*
September 10, 2022	Nerga. Os Castros	Vigo	Gastropods	*Monodonta sp.*
September 10, 2022	Nerga. Os Castros	Vigo	Gastropods	*Patella sp.*
September 10, 2022	Nerga. Os Castros	Vigo	Cnidarias	*n.i.*
September 10, 2022	Nerga. Os Castros	Vigo	Crustaceans	*n.i.*
September 26, 2022	Vilanova de Arousa. Corón	Arousa	Bivalves	*Mytilus galloprovincialis*
September 26, 2022	Vilanova de Arousa. Corón	Arousa	Crustaceans	*n.i.*
September 26, 2022	Vilanova de Arousa. Corón	Arousa	Gastropods	*Littorina sp.*
September 26, 2022	Vilanova de Arousa. Corón	Arousa	Gastropods	*Monodonta sp.*
September 26, 2022	Vilanova de Arousa. Corón	Arousa	Gastropods	*Nucella sp.*
September 26, 2022	Vilanova de Arousa. Corón	Arousa	Gastropods	*Doris verrucosa*
September 26, 2022	Vilanova de Arousa. Corón	Arousa	Gastropods	*Patella sp.*
September 26, 2022	Vilanova de Arousa. Corón	Arousa	Cnidarias	*n.i.*
September 27, 2022	Bueu. Cabo Udra	Pontevedra	Gastropods	*Patella sp.*
September 27, 2022	Porto do Son. Arnela	Muros-Noia	Gastropods	*Patella sp.*
September 27, 2022	Porto do Son. Arnela	Muros-Noia	Echinoderms	*Asterina sp.*
September 27, 2022	Porto do Son. Arnela	Muros-Noia	Polychaetes	*n.i.*
September 27, 2022	Porto do Son. Arnela	Muros-Noia	Gastropods	*Patella sp.*
September 27, 2022	Porto do Son. Arnela	Muros-Noia	Bivalves	*Mytilus galloprovincialis*
September 27, 2022	Porto do Son. Arnela	Muros-Noia	Cnidarias	*n.i.*
September 27, 2022	Porto do Son. Arnela	Muros-Noia	Crustaceans	*n.i.*

September 27, 2022	Porto do Son. Arnela	Muros-Noia	Gastropods	*Aplysia punctata*
September 27, 2022	Porto do Son. Arnela	Muros-Noia	Gastropods	*Monodonta sp.*
September 27, 2022	Porto do Son. Arnela	Muros-Noia	Gastropods	*Gibbula sp.*
September 27, 2022	Porto do Son. Arnela	Muros-Noia	Gastropods	*Nucella sp.*
September 28, 2022	Bueu. Beluso	Pontevedra	Cnidarias	*n.i.*
September 28, 2022	Bueu. Beluso	Pontevedra	Bivalves	*Mytilus galloprovincialis*
September 28, 2022	Bueu. Beluso	Pontevedra	Echinoderms	*Paracentrotus sp.*
September 28, 2022	Bueu. Beluso	Pontevedra	Gastropods	*Crepidula sp.*
September 28, 2022	Bueu. Beluso	Pontevedra	Crustaceans	*n.i.*
September 28, 2022	Bueu. Beluso	Pontevedra	Bivalves	*Ruditapes philippinarum*
September 28, 2022	Bueu. Beluso	Pontevedra	Gastropods	*Nucella sp.*
September 28, 2022	Bueu. Beluso	Pontevedra	Gastropods	*Monodonta sp.*
September 28, 2022	Bueu. Beluso	Pontevedra	Gastropods	*Patella sp.*
September 28, 2022	Aldán. Vilariño	Pontevedra	Bivalves	*Mytilus galloprovincialis*
September 28, 2022	Aldán. Vilariño	Pontevedra	Bivalves	*Ostrea edulis*
September 28, 2022	Aldán. Vilariño	Pontevedra	Bivalves	*Ruditapes philippinarum*
September 28, 2022	Aldán. Vilariño	Pontevedra	Gastropods	*Patella sp.*
September 28, 2022	Aldán. Vilariño	Pontevedra	Crustaceans	*n.i.*
September 28, 2022	Aldán. Vilariño	Pontevedra	Gastropods	*Monodonta sp.*
September 28, 2022	Aldán. Vilariño	Pontevedra	Sea squirts	*n.i.*
September 28, 2022	Aldán. Vilariño	Pontevedra	Gastropods	*Crepidula sp.*
September 28, 2022	Aldán. Vilariño	Pontevedra	Gastropods	*Nucella sp.*
September 28, 2022	Aldán. Vilariño	Pontevedra	Sea squirts	*Phallusia mammillata*
October 5, 2022	Aldán. Vilariño	Pontevedra	Sea squirts	*Ascidiiae indet*
September 29, 2022	O Grove. Pedras negras	Arousa	Cnidarias	*n.i.*
September 28, 2022	Muros. Tal	Muros-Noia	Gastropods	*Littorina sp.*
October 11, 2022	Arousa. Bohido	Arousa	Crustaceans	*Liocarcinus corrugatus*
October 11, 2022	Arousa. Bohido	Arousa	Crustaceans	*Carcinus maenas*
October 11, 2022	Arousa. Bohido	Arousa	Crustaceans	*Liocarcinus arcuatus*

October 17, 2022	Rianxo. Porrón	Arousa	Cnidarias	*n.i.*
July 15, 2022	Corcubión. Pr.Cee	Corcubión	Bivalves	*Cerastoderma edule*
July 20, 2022	Barqueiro. Pr. Salgueira	Barqueiro	Bivalves	*Mytilus galloprovincialis*
July 20, 2022	Barallobre. Rampa	Ferrol	Bivalves	*Mytilus galloprovincialis*
July 19, 2022	Miño. Lombo Espiñeira	Betanzos	Bivalves	*Ruditapes philippinarum*
July 20, 2022	Camariñas. Enseada Vasa	Camariñas	Bivalves	*Ruditapes philippinarum*
July 20, 2022	Corcubión. Pr.Cee	Corcubión	Bivalves	*Mytilus galloprovincialis*
July 19, 2022	Muros I. Cabeiro	Muros-Noia	Bivalves	*Ensis ensis*
July 19, 2022	Pont II. Niño do Corvo	Pontevedra	Bivalves	*Ensis ensis*
July 19, 2022	Pont V. Placeres	Pontevedra	Bivalves	*Ruditapes philippinarum*
July 19, 2022	Vigo II.2. Areiño	Vigo	Bivalves	*Venerupis pullastra*
July 20, 2022	Muros I. Abelleira	Muros-Noia	Bivalves	*Cerastoderma edule*
July 21, 2022	Pont V. Lourizán	Pontevedra	Bivalves	*Ensis ensis*
July 21, 2022	Pont V. Campelo	Pontevedra	Bivalves	*Ruditapes philippinarum*
July 21, 2022	Camariñas. Centro ría	Camariñas	Bivalves	*Ensis siliqua*
July 28, 2022	Barqueiro. Pr. Salgueira	Barqueiro	Bivalves	*Mytilus galloprovincialis*
July 27, 2022	Barallobre. Rampa	Ferrol	Bivalves	*Mytilus galloprovincialis*
July 28, 2022	Miño. Lombo Espiñeira	Betanzos	Bivalves	*Ruditapes philippinarum*
July 27, 2022	Camariñas. Enseada Vasa	Camariñas	Bivalves	*Ruditapes decussatus*
July 27, 2022	Corcubión. Pr.Cee	Corcubión	Bivalves	*Mytilus galloprovincialis*
July 27, 2022	Muros I. Pr.Virxen	Muros-Noia	Bivalves	*Cerastoderma edule*
July 27, 2022	Pont II. Niño do Corvo	Pontevedra	Bivalves	*Ensis ensis*
July 27, 2022	Pont V. Placeres	Pontevedra	Bivalves	*Ruditapes philippinarum*
July 26, 2022	Vigo II.2	Vigo	Bivalves	*Ruditapes philippinarum*
July 27, 2022	Muros I. Pr.Virxen	Muros-Noia	Bivalves	*Ensis ensis*
July 27, 2022	Vigo II.2. Areiño	Vigo	Bivalves	*Ruditapes philippinarum*
July 27, 2022	Vigo II.2. A Guía	Vigo	Bivalves	*Ensis ensis*
August 3, 2022	Barallobre. Rampa	Ferrol	Bivalves	*Mytilus galloprovincialis*
August 2, 2022	Miño. Lombo Espiñeira	Betanzos	Bivalves	*Ruditapes philippinarum*

August 2, 2022	Camariñas. Enseada Vasa	Camariñas	Bivalves	*Ruditapes decussatus*
August 3, 2022	Corcubión. Pr.Cee	Corcubión	Bivalves	*Cerastoderma edule*
August 2, 2022	Muros I. Abelleira	Muros-Noia	Bivalves	*Cerastoderma edule*
August 5, 2022	Muros I	Muros-Noia	Bivalves	*Cerastoderma edule*
August 2, 2022	Pont II. San Cibrao	Pontevedra	Bivalves	*Ruditapes philippinarum*
August 2, 2022	Pont V. Lourizán	Pontevedra	Bivalves	*Ensis ensis*
August 2, 2022	Vigo II.2 Domaio	Vigo	Bivalves	*Ruditapes philippinarum*
August 2, 2022	Pont V. Placeres	Pontevedra	Bivalves	*Ruditapes philippinarum*
August 10, 2022	Barqueiro. Pr. Salgueira	Barqueiro	Bivalves	*Mytilus galloprovincialis*
August 10, 2022	Miño. Lombo Espiñeira	Betanzos	Bivalves	*Ruditapes philippinarum*
August 10, 2022	Camariñas. Paxariñas	Camariñas	Bivalves	*Ruditapes decussatus*
August 9, 2022	Corcubión. Pr.Cee	Corcubión	Bivalves	*Mytilus galloprovincialis*
August 8, 2022	Muros I. Abelleira	Muros-Noia	Bivalves	*Cerastoderma edule*
August 9, 2022	Pont V. Placeres	Pontevedra	Bivalves	*Ruditapes philippinarum*
August 9, 2022	Vigo II.2 Xunqueira	Vigo	Bivalves	*Ruditapes philippinarum*
August 18, 2022	Barqueiro. Pr. Salgueira	Barqueiro	Bivalves	*Mytilus galloprovincialis*
August 18, 2022	Barallobre. Pantalán	Ferrol	Bivalves	*Mytilus galloprovincialis*
August 17, 2022	Miño. Lombo Espiñeira	Betanzos	Bivalves	*Ruditapes philippinarum*
August 18, 2022	Camariñas. Paxariñas	Camariñas	Bivalves	*Ruditapes philippinarum*
August 18, 2022	Corcubión. Pr.Cee	Corcubión	Bivalves	*Cerastoderma edule*
August 17, 2022	Muros I. Abelleira	Muros-Noia	Bivalves	*Cerastoderma edule*
August 18, 2022	Arousa I. Moreiras	Arousa	Bivalves	*Ruditapes philippinarum*
August 19, 2022	Pont V. Placeres	Pontevedra	Bivalves	*Ruditapes philippinarum*
August 18, 2022	Vigo II.2. Areiño	Vigo	Bivalves	*Venerupis pullastra*
August 19, 2022	Barallobre. Centro ría	Ferrol	Bivalves	*Venerupis rhomboideus*
August 26, 2022	Barqueiro. Pr. Salgueira	Barqueiro	Bivalves	*Mytilus galloprovincialis*
August 23, 2022	Barallobre. Pr.Maniños	Ferrol	Bivalves	*Cerastoderma edule*
August 24, 2022	Miño. Lombo Esp.	Betanzos	Bivalves	*Ruditapes philippinarum*
August 23, 2022	Camariñas. Pasariña	Camariñas	Bivalves	*Ruditapes philippinarum*

August 24, 2022	Corcubión. Pr.Cee	Corcubión	Bivalves	*Cerastoderma edule*
August 23, 2022	Muros I. Pr.Virxen	Muros-Noia	Bivalves	*Cerastoderma edule*
August 25, 2022	Arousa I. Moreiras	Arousa	Bivalves	*Ruditapes philippinarum*
August 22, 2022	Pont V. Placeres	Pontevedra	Bivalves	*Ruditapes philippinarum*
August 24, 2022	Vigo II.2. ETEA	Vigo	Bivalves	*Ruditapes philippinarum*
August 31, 2022	Barqueiro. Pr. Salgueira	Barqueiro	Bivalves	*Mytilus galloprovincialis*
August 30, 2022	Barallobre. Maniños	Ferrol	Bivalves	*Ruditapes philippinarum*
August 30, 2022	Miño. Lombo Esp.	Betanzos	Bivalves	*Ruditapes philippinarum*
August 30, 2022	Camariñas. Pasariña	Camariñas	Bivalves	*Ruditapes decussatus*
August 30, 2022	Corcubión. Pr.Cee	Corcubión	Bivalves	*Mytilus galloprovincialis*
August 30, 2022	Muros I. Abelleira	Muros-Noia	Bivalves	*Cerastoderma edule*
August 30, 2022	Arousa I. Moreiras	Arousa	Bivalves	*Ruditapes philippinarum*
August 30, 2022	Pont V. Placeres	Pontevedra	Bivalves	*Ruditapes philippinarum*
August 30, 2022	Vigo II.2. Pr.Xunqueira	Vigo	Bivalves	*Ruditapes philippinarum*
September 7, 2022	Barqueiro. Pr. Salgueira	Barqueiro	Bivalves	*Mytilus galloprovincialis*
September 6, 2022	Barallobre. Pt del Tren	Ferrol	Bivalves	*Ruditapes philippinarum*
September 7, 2022	Miño. Lombo Esp.	Betanzos	Bivalves	*Ruditapes philippinarum*
September 7, 2022	Camariñas. Zona E	Camariñas	Bivalves	*Ensis ensis*
September 6, 2022	Corcubión. Pr.Cee	Corcubión	Bivalves	*Mytilus galloprovincialis*
September 7, 2022	Muros I. Pr.Virxen	Muros-Noia	Bivalves	*Cerastoderma edule*
September 7, 2022	Muros III. Testal	Muros-Noia	Bivalves	*Cerastoderma edule*
September 7, 2022	Arousa I. Pr.Carreiro	Arousa	Bivalves	*Ensis ensis*
September 8, 2022	Pont II. San Cibrao	Pontevedra	Bivalves	*Cerastoderma edule*
September 8, 2022	Pont V. Placeres	Pontevedra	Bivalves	*Ruditapes philippinarum*
September 8, 2022	Vigo II.2. ETEA	Vigo	Bivalves	*Ruditapes philippinarum*
September 7, 2022	Camariñas. Pasariña	Camariñas	Bivalves	*Ruditapes decussatus*
September 8, 2022	Vigo II.2. A Guia	Vigo	Bivalves	*Ensis ensis*
September 9, 2022	Pont II	Pontevedra	Bivalves	*Ensis ensis*
September 13, 2022	Barqueiro. Pr. Salgueira	Barqueiro	Bivalves	*Mytilus galloprovincialis*

September 14, 2022	Barallobre. Pantalán	Ferrol	Bivalves	*Mytilus galloprovincialis*
September 13, 2022	Miño. Lombo Esp	Betanzos	Bivalves	*Ruditapes philippinarum*
September 14, 2022	Camariñas. Río da Ponte	Camariñas	Bivalves	*Ruditapes decussatus*
September 13, 2022	Corcubión. Pr.Cee	Corcubión	Bivalves	*Mytilus galloprovincialis*
September 14, 2022	Muros I. Pr.Virxen	Muros-Noia	Bivalves	*Cerastoderma edule*
September 13, 2022	Muros III. Testal	Muros-Noia	Bivalves	*Cerastoderma edule*
September 14, 2022	Arousa I. Cantodorxo	Arousa	Bivalves	*Ruditapes philippinarum*
September 14, 2022	Arousa VI. Sarrido	Arousa	Bivalves	*Ruditapes philippinarum*
September 13, 2022	Pont II. San Cibrao	Pontevedra	Bivalves	*Cerastoderma edule*
September 16, 2022	Pont V. Placeres	Pontevedra	Bivalves	*Ruditapes philippinarum*
September 15, 2022	Vigo II.2. ETEA	Vigo	Bivalves	*Ruditapes decussatus*
September 16, 2022	Muros I. Pr.Virxen	Muros-Noia	Bivalves	*Cerastoderma edule*
September 16, 2022	Muros III. Testal	Muros-Noia	Bivalves	*Cerastoderma edule*
September 21, 2022	Barqueiro. Pr. Salgueira	Barqueiro	Bivalves	*Mytilus galloprovincialis*
September 22, 2022	Miño. Lombo Esp.	Betanzos	Bivalves	*Ruditapes philippinarum*
September 21, 2022	Camariñas. Río da Ponte	Camariñas	Bivalves	*Ruditapes philippinarum*
September 21, 2022	Corcubión. Pr.Cee	Corcubión	Bivalves	*Mytilus galloprovincialis*
September 20, 2022	Muros I. Abelleira	Muros-Noia	Bivalves	*Cerastoderma edule*
September 20, 2022	Muros III. Testal	Muros-Noia	Bivalves	*Cerastoderma edule*
September 20, 2022	Arousa I. Meloxo	Arousa	Bivalves	*Venerupis pullastra*
September 20, 2022	Pont II. San Cibrao	Pontevedra	Bivalves	*Cerastoderma edule*
September 20, 2022	Pont V. Placeres	Pontevedra	Bivalves	*Ruditapes philippinarum*
September 21, 2022	Vigo II.2. ETEA	Vigo	Bivalves	*Ruditapes philippinarum*
September 21, 2022	Vigo II.2. A Guia	Vigo	Bivalves	*Ensis ensis*
September 21, 2022	Camariñas. Zona E	Camariñas	Bivalves	*Ensis ensis*
September 23, 2022	Muros I. Pr.Virxen	Muros-Noia	Bivalves	*Cerastoderma edule*
September 23, 2022	Muros III. Testal	Muros-Noia	Bivalves	*Cerastoderma edule*
September 23, 2022	Arousa I. Airo grande	Arousa	Bivalves	*Ruditapes philippinarum*
September 27, 2022	Barqueiro. Pr. Salgueira	Barqueiro	Bivalves	*Mytilus galloprovincialis*

September 28, 2022	Barallobre. Pr.Maniños	Ferrol	Bivalves	*Cerastoderma edule*
September 27, 2022	Miño. Lombo Esp.	Betanzos	Bivalves	*Ruditapes philippinarum*
September 27, 2022	Camariñas. Pasariña	Camariñas	Bivalves	*Ruditapes decussatus*
September 27, 2022	Corcubión. Pr.Cee	Corcubión	Bivalves	*Ensis ensis*
September 27, 2022	Muros I. Pta.Cabeiro	Muros-Noia	Bivalves	*Ensis ensis*
September 26, 2022	Muros III	Muros-Noia	Bivalves	*Cerastoderma edule*
September 27, 2022	Pont II. Niño do corvo	Pontevedra	Bivalves	*Ensis ensis*
September 27, 2022	Pont V. Placeres	Pontevedra	Bivalves	*Ruditapes philippinarum*
September 27, 2022	Vigo II.2. ETEA	Vigo	Bivalves	*Ruditapes philippinarum*
September 27, 2022	Vigo II.2	Vigo	Bivalves	*Ensis ensis*
September 28, 2022	Muros III	Muros-Noia	Bivalves	*Ruditapes philippinarum*
September 28, 2022	Muros III	Muros-Noia	Bivalves	*Cerastoderma edule*
September 29, 2022	Vigo II.2	Vigo	Bivalves	*Ruditapes philippinarum*
September 30, 2022	Pont V. Placeres	Pontevedra	Bivalves	*Ruditapes philippinarum*
October 5, 2022	Barqueiro. Pr. Salgueira	Barqueiro	Bivalves	*Mytilus galloprovincialis*
October 4, 2022	Barallobre. Pantalán	Ferrol	Bivalves	*Mytilus galloprovincialis*
October 5, 2022	Miño. Lombo Esp.	Betanzos	Bivalves	*Ruditapes philippinarum*
October 4, 2022	Camariñas. Pasariña	Camariñas	Bivalves	*Ruditapes decussatus*
October 5, 2022	Corcubión. Pr.Cee	Corcubión	Bivalves	*Cerastoderma edule*
October 4, 2022	Muros I. Pr.Virxen	Muros-Noia	Bivalves	*Ensis ensis*
October 3, 2022	Muros III. Pedras	Muros-Noia	Bivalves	*Ruditapes philippinarum*
October 4, 2022	Pont II. Sartaxéns	Pontevedra	Bivalves	*Ensis ensis*
October 4, 2022	Pont V. Placeres	Pontevedra	Bivalves	*Ruditapes philippinarum*
October 4, 2022	Vigo II.2 ETEA	Vigo	Bivalves	*Ruditapes philippinarum*
October 4, 2022	Camariñas. Zona E	Camariñas	Bivalves	*Ensis ensis*
October 4, 2022	Pont II. Río Esteiro	Pontevedra	Bivalves	*Cerastoderma edule*
October 4, 2022	Vigo II.2. A Guía	Vigo	Bivalves	*Ensis ensis*
October 5, 2022	Muros III. As pedras	Muros-Noia	Bivalves	*Ruditapes philippinarum*
October 5, 2022	Corcubión. Pr.Cee	Corcubión	Bivalves	*Ensis ensis*

October 5, 2022	Muros III. As pedras	Muros-Noia	Bivalves	*Cerastoderma edule*
October 5, 2022	Camariñas. Pasariña	Camariñas	Bivalves	*Ruditapes decussatus*
October 5, 2022	Camariñas. Ria Camariña	Camariñas	Bivalves	*Ensis ensis*
October 11, 2022	Barqueiro. Pr. Salgueira	Barqueiro	Bivalves	*Mytilus galloprovincialis*
October 11, 2022	Barallobre. Pr.Maniños	Ferrol	Bivalves	*Ruditapes philippinarum*
October 11, 2022	Miño. Lombo Esp.	Betanzos	Bivalves	*Cerastoderma edule*
October 11, 2022	Camariñas. Pasariña	Camariñas	Bivalves	*Ruditapes decussatus*
October 11, 2022	Corcubión. Pr.Cee	Corcubión	Bivalves	*Cerastoderma edule*
October 11, 2022	Muros I. Cabeiro	Muros-Noia	Bivalves	*Ensis ensis*
October 10, 2022	Muros III. Testal	Muros-Noia	Bivalves	*Cerastoderma edule*
October 11, 2022	Pont V. Placeres	Pontevedra	Bivalves	*Ensis ensis*
October 10, 2022	Muros III. Testal	Muros-Noia	Bivalves	*Ruditapes philippinarum*
October 11, 2022	Pont V. Placeres	Pontevedra	Bivalves	*Ruditapes philippinarum*
October 19, 2022	Barqueiro. Pr. Salgueira	Barqueiro	Bivalves	*Mytilus galloprovincialis*
October 18, 2022	Barallobre. Pr.Maniños	Ferrol	Bivalves	*Ruditapes philippinarum*
October 18, 2022	Miño. Lombo Esp.	Betanzos	Bivalves	*Cerastoderma edule*
October 18, 2022	Camariñas. Paxariña	Camariñas	Bivalves	*Ruditapes decussatus*
October 19, 2022	Corcubión. Pr.Cee	Corcubión	Bivalves	*Cerastoderma edule*
October 17, 2022	Muros III. Testal	Muros-Noia	Bivalves	*Ruditapes philippinarum*
October 18, 2022	Pont II. Area Brava	Pontevedra	Bivalves	*Ensis ensis*
October 17, 2022	Muros III. Testal	Muros-Noia	Bivalves	*Cerastoderma edule*
October 19, 2022	Po II. Aldan	Pontevedra	Bivalves	*Cerastoderma edule*
October 26, 2022	Barqueiro	Barqueiro	Bivalves	*Mytilus galloprovincialis*
October 25, 2022	Barallobre. Pantalán	Ferrol	Bivalves	*Mytilus galloprovincialis*
October 25, 2022	Miño. Lombo Esp.	Betanzos	Bivalves	*Cerastoderma edule*
October 25, 2022	Camariñas. Paxariña	Camariñas	Bivalves	*Ruditapes decussatus*
October 25, 2022	Corcubión. Pr.Cee	Corcubión	Bivalves	*Cerastoderma edule*
October 25, 2022	Muros I. Cabaneiro	Muros-Noia	Bivalves	*Ensis ensis*
October 24, 2022	Muros III. Testal	Muros-Noia	Bivalves	*Cerastoderma edule*

November 4, 2022	Barqueiro. Pr. Salgueira	Barqueiro	Bivalves	*Mytilus galloprovincialis*
November 3, 2022	Miño. Lombo Esp.	Betanzos	Bivalves	*Cerastoderma edule*
November 3, 2022	Camariñas. Paxariña	Camariñas	Bivalves	*Ruditapes decussatus*
November 2, 2022	Corcubión. Pr.Cee	Corcubión	Bivalves	*Mytilus galloprovincialis*
November 2, 2022	Muros III. Testal	Muros-Noia	Bivalves	*Cerastoderma edule*
November 2, 2022	Pont II. Cofeife	Pontevedra	Bivalves	*Ensis ensis*
November 4, 2022	Pont V. Placeres	Pontevedra	Bivalves	*Ruditapes philippinarum*
November 4, 2022	Pont II. Rio Esteiro	Pontevedra	Bivalves	*Cerastoderma edule*
November 8, 2022	Barqueiro. Pr. Salgueira	Barqueiro	Bivalves	*Mytilus galloprovincialis*
November 8, 2022	Miño. Lombo Esp	Betanzos	Bivalves	*Cerastoderma edule*
November 8, 2022	Camariñas. Paxariña	Camariñas	Bivalves	*Ruditapes decussatus*
November 8, 2022	Corcubión. Pr.Cee	Corcubión	Bivalves	*Mytilus galloprovincialis*
November 7, 2022	Muros III	Muros-Noia	Bivalves	*Ruditapes philippinarum*
November 11, 2022	Pont V. Placeres	Pontevedra	Bivalves	*Ruditapes philippinarum*
November 15, 2022	Barallobre. Rampa	Ferrol	Bivalves	*Mytilus galloprovincialis*
November 15, 2022	Miño. Lombo Esp	Betanzos	Bivalves	*Cerastoderma edule*
November 15, 2022	Camariñas. Rio da Ponte	Camariñas	Bivalves	*Ruditapes decussatus*
November 15, 2022	Muros I. Cabaneiro	Muros-Noia	Echinoderms	*Holoturia sp.*
November 25, 2022	Porto do Son. Arnela	Muros-Noia	Echinoderms	*Asterina sp.*
November 25, 2022	Porto do Son. Arnela	Muros-Noia	Echinoderms	*Paracentrotus sp.*
November 25, 2022	Porto do Son. Arnela	Muros-Noia	Cnidarias	*n.i.*
November 25, 2022	Porto do Son. Arnela	Muros-Noia	Crustaceans	*n.i.*
November 25, 2022	Porto do Son. Arnela	Muros-Noia	Bivalves	*Mytilus galloprovincialis*
November 25, 2022	Porto do Son. Arnela	Muros-Noia	Gastropods	*Monodonta sp.*
November 25, 2022	Porto do Son. Arnela	Muros-Noia	Gastropods	*Patella sp.*
November 25, 2022	Porto do Son. Arnela	Muros-Noia	Cnidarias	*n.i.*
November 25, 2022	Porto do Son. Arnela	Muros-Noia	Gastropods	*Nucella sp.*
November 25, 2022	Ramallosa. Esteiro Foz	Vigo	Bivalves	*Ruditapes philippinarum*
November 25, 2022	Ramallosa. Esteiro Foz	Vigo	Bivalves	*Mytilus galloprovincialis*

November 25, 2022	Ramallosa. Esteiro Foz	Vigo	Bivalves	*Ostrea edulis*
November 25, 2022	Ramallosa. Esteiro Foz	Vigo	Gastropods	*Littorina sp.*
November 25, 2022	Ramallosa. Esteiro Foz	Vigo	Crustaceans	*Polybius sp.*
November 25, 2022	Bueu. Beluso	Pontevedra	Echinoderms	*Paracentrotus sp.*
November 25, 2022	Bueu. Beluso	Pontevedra	Bivalves	*Mytilus galloprovincialis*
November 25, 2022	Bueu. Beluso	Pontevedra	Gastropods	*Patella sp.*
November 25, 2022	Bueu. Beluso	Pontevedra	Cnidarias	*n.i.*
November 25, 2022	Bueu. Beluso	Pontevedra	Gastropods	*Monodonta sp.*
November 25, 2022	Bueu. Beluso	Pontevedra	Gastropods	*Gibbula sp.*
November 28, 2022	Aldán. Vilariño	Pontevedra	Gastropods	*Patella sp.*
November 28, 2022	Aldán. Vilariño	Pontevedra	Bivalves	*Dosinia exoleta*
November 28, 2022	Aldán. Vilariño	Pontevedra	Gastropods	*Nucella sp.*
November 28, 2022	Aldán. Vilariño	Pontevedra	Gastropods	*Monodonta sp.*
November 28, 2022	Aldán. Vilariño	Pontevedra	Bivalves	*Ostrea edulis*
November 28, 2022	Aldán. Vilariño	Pontevedra	Cnidarias	*n.i.*
November 28, 2022	Aldán. Vilariño	Pontevedra	Bivalves	*Mytilus galloprovincialis*
November 10, 2022	Arousa. Bohido	Arousa	Crustaceans	*Inachus phalangium*
November 10, 2022	Arousa. Bohido	Arousa	Crustaceans	*Liocarcinus arcuatus*
November 10, 2022	Arousa. Bohido	Arousa	Crustaceans	*Liocarcinus corrugatus*
November 10, 2022	Arousa. Bohido	Arousa	Crustaceans	*Atelecyclus undecimdentatus*
November 10, 2022	Arousa. Bohido	Arousa	Crustaceans	*Pagurus prideaux*
November 10, 2022	Arousa. Bohido	Arousa	Echinoderms	*Asterina gibbosa*
November 10, 2022	Arousa. Bohido	Arousa	Gastropods	*Tritia reticulata*
November 10, 2022	Muros	Muros-Noia	Crustaceans	*Polybius sp.*
October 25, 2022	Cangas	Vigo	Bivalves	*Ruditapes decussatus*
November 16, 2022	Barqueiro. Pr. Salgueira	Barqueiro	Bivalves	*Mytilus galloprovincialis*
November 16, 2022	Corcubión. Pr.Cee	Corcubión	Bivalves	*Mytilus galloprovincialis*
November 23, 2022	Barqueiro. Pr. Salgueira	Barqueiro	Bivalves	*Mytilus galloprovincialis*
November 22, 2022	Barallobre. Pantalán	Ferrol	Bivalves	*Mytilus galloprovincialis*

November 22, 2022	Miño. Lombo Esp.	Betanzos	Bivalves	*Cerastoderma edule*
November 22, 2022	Pasaxe. O Parrote	A Coruña	Bivalves	*Mytilus galloprovincialis*
November 22, 2022	Camariñas. Paxariña	Camariñas	Bivalves	*Ruditapes decussatus*
November 22, 2022	Corcubión. Pr.Cee	Corcubión	Bivalves	*Mytilus galloprovincialis*
November 29, 2022	Barqueiro. Pr. Salgueira	Barqueiro	Bivalves	*Mytilus galloprovincialis*
November 29, 2022	Barallobre. Pantalán	Ferrol	Bivalves	*Mytilus galloprovincialis*
November 29, 2022	Miño. Muro Petra Sabio	Betanzos	Bivalves	*Mytilus galloprovincialis*
November 29, 2022	Pasaxe. O Parrote	A Coruña	Bivalves	*Mytilus galloprovincialis*
November 29, 2022	Camariñas. Paxariña	Camariñas	Bivalves	*Ruditapes decussatus*
November 30, 2022	Corcubión. Pr.Cee	Corcubión	Bivalves	*Mytilus galloprovincialis*
December 7, 2022	Barqueiro. Pr. Salgueira	Barqueiro	Bivalves	*Mytilus galloprovincialis*
December 7, 2022	Miño. Muro Petra Sabio	Betanzos	Bivalves	*Mytilus galloprovincialis*
December 7, 2022	Camariñas. Enseada Vasa	Camariñas	Bivalves	*Mytilus galloprovincialis*
December 7, 2022	Corcubión. Pr.Cee	Corcubión	Bivalves	*Mytilus galloprovincialis*
December 13, 2022	Barqueiro. Pr. Salgueira	Barqueiro	Bivalves	*Mytilus galloprovincialis*
December 13, 2022	Barallobre. Rampa	Ferrol	Bivalves	*Mytilus galloprovincialis*
December 13, 2022	Miño. Muro Petra Sabio	Betanzos	Bivalves	*Mytilus galloprovincialis*
December 13, 2022	Camariñas. Río da Ponte	Camariñas	Bivalves	*Ruditapes decussatus*
December 13, 2022	Corcubión. Pr.Cee	Corcubión	Bivalves	*Mytilus galloprovincialis*
December 21, 2022	Barqueiro. Pr. Salgueira	Barqueiro	Bivalves	*Mytilus galloprovincialis*
December 20, 2022	Barallobre. Rampa	Ferrol	Bivalves	*Mytilus galloprovincialis*
December 21, 2022	Miño. Muro Petra Sabio	Betanzos	Bivalves	*Mytilus galloprovincialis*
December 22, 2022	Camariñas. Paxariña	Camariñas	Bivalves	*Ruditapes decussatus*
December 20, 2022	Corcubión. Pr.Cee	Corcubión	Bivalves	*Mytilus galloprovincialis*
December 27, 2022	Barqueiro. Pr. Salgueira	Barqueiro	Bivalves	*Mytilus galloprovincialis*
December 27, 2022	Barallobre. Pantalán	Ferrol	Bivalves	*Mytilus galloprovincialis*
December 27, 2022	Miño. Muro Petra Sabio	Betanzos	Bivalves	*Mytilus galloprovincialis*
December 27, 2022	Camariñas. Río da Ponte	Camariñas	Bivalves	*Ruditapes decussatus*
April 6, 2021	Vigo. Cabo Estai	Vigo	Echinoderms	*Paracentrotus lividus*

April 6, 2021	Vigo. Cabo Estai	Vigo	Gastropods	*Monodonta lineata*
April 6, 2021	Vigo. Cabo Estai	Vigo	Gastropods	*Patella spp.*
April 6, 2021	Vigo. Cabo Estai	Vigo	Cnidarias	*Actinia equina*
April 6, 2021	Vigo. Cabo Estai	Vigo	Crustaceans	*Pachygrapsus marmoratus*
April 6, 2021	Vigo. Cabo Estai	Vigo	Cnidarias	*Anemonia viridis*
May 25, 2021	Vigo. Praia A Fontaíña	Vigo	Gastropods	*Patella spp.*
May 25, 2021	Vigo. Praia A Fontaíña	Vigo	Echinoderms	*Marthasterias glacialis*
May 25, 2021	Vigo. Praia A Fontaíña	Vigo	Cnidarias	*Actinia equina*
May 25, 2021	Vigo. Praia A Fontaíña	Vigo	Echinoderms	*Paracentrotus lividus*
May 25, 2021	Vigo. Praia Canido	Vigo	Echinoderms	*Paracentrotus lividus*
May 25, 2021	Vigo. Praia Canido	Vigo	Cnidarias	*Actinia equina*
May 25, 2021	Vigo. Praia Canido	Vigo	Gastropods	*Haliotis tuberculata*
May 25, 2021	Vigo. Praia Canido	Vigo	Gastropods	*Patella spp.*
May 25, 2021	Vigo. Praia Canido	Vigo	Cnidarias	*Anemonia viridis*
May 25, 2021	Vigo. Praia Canido	Vigo	Gastropods	*Monodonta lineata*
June 6, 2021	Vigo. Monteferro	Vigo	Cnidarias	*Actinia equina*
June 6, 2021	Vigo. Monteferro	Vigo	Cnidarias	*Anemonia viridis*
June 6, 2021	Vigo. Monteferro	Vigo	Gastropods	*Monodonta lineata*
June 6, 2021	Vigo. Monteferro	Vigo	Gastropods	*Patella spp.*
June 6, 2021	Vigo. Monteferro	Vigo	Echinoderms	*Paracentrotus lividus*
June 6, 2021	Vigo. Monteferro	Vigo	Gastropods	*Haliotis tuberculata*
June 6, 2021	Vigo. Monteferro	Vigo	Crustaceans	*Cancer pagurus*
June 6, 2021	Vigo. Monteferro	Vigo	Crustaceans	*Pachygrapsus marmoratus*
June 6, 2021	Vigo. Monteferro	Vigo	Crustaceans	*Carcinus maenas*
June 24, 2021	Baiona. Cabo Silleiro	Vigo	Gastropods	*Monodonta lineata*
June 24, 2021	Baiona. Cabo Silleiro	Vigo	Gastropods	*Patella spp.*
June 24, 2021	Baiona. Cabo Silleiro	Vigo	Echinoderms	*Paracentrotus lividus*
June 24, 2021	Baiona. Cabo Silleiro	Vigo	Echinoderms	*Marthasterias glacialis*
June 24, 2021	Baiona. Cabo Silleiro	Vigo	Cnidarias	*Actinia equina*

June 24, 2021	Baiona. Cabo Silleiro	Vigo	Cnidarias	*Anemonia viridis*
June 24, 2021	Baiona. Cabo Silleiro	Vigo	Crustaceans	*Pachygrapsus marmoratus*
September 10, 2021	Vigo. Porto	Vigo	Cnidarias	*Veretillum cynomorium*
September 12, 2021	Sada. Porto	Betanzos	Gastropods	*Nassarius sp.*
October 6, 2021	Vigo. Praia Canido	Vigo	Cnidarias	*Anemonia viridis*
October 6, 2021	Vigo. Praia Canido	Vigo	Crustaceans	*Necora puber*
October 6, 2021	Vigo. Praia Canido	Vigo	Crustaceans	*Palaemon serratus*
October 6, 2021	Vigo. Praia Canido	Vigo	Echinoderms	*Paracentrotus lividus*
September 3, 2021	41.912, -9.1965[*]	Vigo	Gastropods	*Neptunea contraria*
September 5, 2021	42.3216, -9.067[*]	Vigo	Echinoderms	*Luidia sarsi*
September 2, 2021	42.0436, -9.1135[*]	Vigo	Cephalopods	*Todaropsis eblanae*
September 2, 2021	42.0436, -9.1135[*]	Vigo	Cephalopods	*Todaropsis eblanae*
September 2, 2021	42.0436, -9.1135[*]	Vigo	Cephalopods	*Eledone cirrhosa*
September 3, 2021	41.9055, -9.076[*]	Vigo	Cephalopods	*Alloteuthis sp.*
September 3, 2021	41.912, -9.1965[*]	Vigo	Gastropods	*Buccinum humphreysianum*
September 3, 2021	41.912, -9.1965[*]	Vigo	Crustaceans	*Macropipus tuberculatus*
September 11, 2021	42.7436, -9.3263[*]	Muros-Noia	Polychaetes	*Aphrodite aculeata*
September 8, 2021	42.3438, -9.093[*]	Pontevedra	Polychaetes	*Aphrodite aculeata*
September 3, 2021	41.9055, -9.076[*]	Vigo	Sea squirts	*Corella paralelograma*
September 3, 2021	41.912, -9.1965[*]	Vigo	Gastropods	*Colus gracilis*
September 3, 2021	41.9045, -9.0748[*]	Vigo	Crustaceans	*Munida intermedia*
September 2, 2021	42.0436, -9.1135[*]	Vigo	Crustaceans	*Pagurus prideaux*
September 2, 2021	42.0436, -9.1135[*]	Vigo	Echinoderms	*Parastichopus regalis*
September 5, 2021	42.231, -9.068[*]	Vigo	Echinoderms	*Brissopsis lyrifera*
September 8, 2021	42.2856, -9.1486[*]	Vigo	Cnidarias	*Actinauge richardi*
September 8, 2021	42.2856, -9.14866[*]	Vigo	Gastropods	*Euspira fusca*
October 25, 2021	Vigo. Porto	Vigo	Cephalopods	*Sepia officinalis*
October 25, 2021	Vigo. Porto	Vigo	Cephalopods	*Sepia officinalis*
October 25, 2021	Vigo. Porto	Vigo	Cnidarias	*Veretillum cynomorium*

September 5, 2021	42.231, -9.068*	Vigo	Cnidarias	*Funiculina quadrangularis*
September 3, 2021	41.9055, -9.076*	Vigo	Cnidarias	*Adamsia carcinopadus*
April 6, 2021	Vigo. Cabo Estai	Vigo	Crustaceans	*Palaemon serratus*
May 25, 2021	Vigo. Praia A Fontaíña	Vigo	Bivalves	*Mytilus galloprovincialis*
May 25, 2021	Vigo. Praia Canido	Vigo	Bivalves	*Mytilus galloprovincialis*
June 24, 2021	Baiona. Cabo Silleiro	Vigo	Bivalves	*Mytilus galloprovincialis*
June 24, 2021	Baiona. Cabo Silleiro	Vigo	Crustaceans	*Pollicipes pollicipes*
September 12, 2021	IEO. Sada. Porto	Betanzos	Bivalves	*Ostrea edulis*
October 6, 2021	Vigo. Praia Canido	Vigo	Bivalves	*Mytilus galloprovincialis*
October 6, 2021	Vigo. Praia Canido	Vigo	Gastropods	*Haliotis tuberculata*
October 6, 2021	Vigo. Praia Canido	Vigo	Gastropods	*Patella vulgata*
October 6, 2021	Vigo. Praia Canido	Vigo	Gastropods	*Monodonta lineata*
September 2, 2021	42.0436, -9.1135*	Vigo	Cephalopods	*Eledone cirrhosa*
September 3, 2021	41.9055, -9.076*	Vigo	Crustaceans	*Pagurus excavatus*
September 2, 2021	42.0436, -9.1135*	Vigo	Crustaceans	*Polybius henslowii*
September 3, 2021	41.9055, -9.076*	Vigo	Gastropods	*Neptunea contraria*
September 2, 2021	42.0436, -9.1135*	Vigo	Cephalopods	*Illex coindetti*
February 3, 2022	Vigo. Praia de Samil	Vigo	Crustaceans	*Polybius henslowii*
June 16, 2022	Vigo. Praia Canido	Vigo	Echinoderms	*Paracentrotus lividus*
June 16, 2022	Vigo. Praia Canido	Vigo	Crustaceans	*Eriphia verrucosa*
June 16, 2022	Vigo. Praia Canido	Vigo	Crustaceans	*Necora puber*
June 16, 2022	Vigo. Praia Canido	Vigo	Bivalves	*Mytilus galloprovincialis*
June 16, 2022	Vigo. Praia Canido	Vigo	Gastropods	*Patella spp.*
June 16, 2022	Vigo. Praia Canido	Vigo	Gastropods	*Haliotis tuberculata*
June 16, 2022	Vigo. Praia Canido	Vigo	Crustaceans	*Pollicipes pollicipes*
June 16, 2022	Vigo. Praia Canido	Vigo	Gastropods	*Monodonta lineata*
June 16, 2022	Vigo. Praia Canido	Vigo	Cnidarias	*Anemonia viridis*
June 27, 2022	Vigo. Monteferro	Vigo	Gastropods	*Patella spp.*
June 27, 2022	Vigo. Monteferro	Vigo	Gastropods	*Monodonta lineata*

June 27, 2022	Vigo. Monteferro	Vigo	Cnidarias	*Anemonia viridis*
June 27, 2022	Vigo. Monteferro	Vigo	Crustaceans	*Necora puber*
June 27, 2022	Vigo. Monteferro	Vigo	Crustaceans	*Eriphia verrucosa*
June 27, 2022	Vigo. Monteferro	Vigo	Crustaceans	*Maja squinado*
July 12, 2022	Vigo. Cabo Estai	Vigo	Gastropods	*Monodonta lineata*
July 12, 2022	Vigo. Cabo Estai	Vigo	Gastropods	*Patella spp.*
July 12, 2022	Vigo. Cabo Estai	Vigo	Echinoderms	*Paracentrotus lividus*
July 12, 2022	Vigo. Cabo Estai	Vigo	Crustaceans	*Eriphia verrucosa*
July 12, 2022	Vigo. Cabo Estai	Vigo	Crustaceans	*Palaemon serratus*
July 12, 2022	Vigo. Cabo Estai	Vigo	Cnidarias	*Anemonia viridis*
July 12, 2022	A Coruña. Praia da Cruz	A Coruña	Echinoderms	*Paracentrotus lividus*
July 12, 2022	A Coruña. Praia da Cruz	A Coruña	Bivalves	*Mytilus galloprovincialis*
July 12, 2022	A Coruña. Praia da Cruz	A Coruña	Gastropods	*Patella spp.*
July 12, 2022	A Coruña. Praia da Cruz	A Coruña	Gastropods	*Monodonta lineata*
July 28, 2022	A Coruña. Praia de Oza	A Coruña	Bivalves	*Mytilus galloprovincialis*
July 28, 2022	A Coruña. Praia de Oza	A Coruña	Bivalves	*Ostrea edulis*
August 11, 2022	Mougás. Praia Pedra Rubia	Vigo	Gastropods	*Haliotis tuberculata*
August 11, 2022	Mougás. Praia Pedra Rubia	Vigo	Crustaceans	*Palaemon serratus*
August 11, 2022	Mougás. Praia Pedra Rubia	Vigo	Echinoderms	*Paracentrotus lividus*
August 11, 2022	Mougás. Praia Pedra Rubia	Vigo	Echinoderms	*Aplysia*
August 11, 2022	Mougás. Praia Pedra Rubia	Vigo	Cnidarias	*Anemonia viridis*
August 11, 2022	Mougás. Praia Pedra Rubia	Vigo	Crustaceans	*Pollicipes pollicipes*
August 11, 2022	Mougás. Praia Pedra Rubia	Vigo	Gastropods	*Patella spp.*
August 11, 2022	Mougás. Praia Pedra Rubia	Vigo	Gastropods	*Monodonta lineata*
August 11, 2022	Mougás. Praia Pedra Rubia	Vigo	Bivalves	*Mytilus galloprovincialis*
August 11, 2022	Mougás. Praia Pedra Rubia	Vigo	Echinoderms	*Asterina spp.*
August 26, 2022	Baiona. Cabo Silleiro	Vigo	Crustaceans	*Pollicipes pollicipes*
August 26, 2022	Baiona. Cabo Silleiro	Vigo	Echinoderms	*Paracentrotus lividus*
August 26, 2022	Baiona. Cabo Silleiro	Vigo	Gastropods	*Patella spp.*

August 26, 2022	Baiona. Cabo Silleiro	Vigo	Echinoderms	*Asterina spp.*
August 26, 2022	Ramallosa. Esteiro Foz	Vigo	Bivalves	*Mytilus galloprovincialis*
August 26, 2022	Ramallosa. Esteiro Foz	Vigo	Bivalves	*Ruditapes decussatus*
August 26, 2022	Ramallosa. Esteiro Foz	Vigo	Bivalves	*Cardiidae*
August 26, 2022	Ramallosa. Esteiro Foz	Vigo	Crustaceans	*Necora puber*
September 1, 2022	Cangas. Punta Balea	Vigo	Crustaceans	*Palaemon serratus*
September 1, 2022	Cangas. Punta Balea	Vigo	Echinoderms	*Paracentrotus lividus*
September 1, 2022	Cangas. Punta Balea	Vigo	Gastropods	*Monodonta lineata*
September 1, 2022	Cangas. Punta Balea	Vigo	Bivalves	*Venus verrucosa*
September 1, 2022	Cangas. Punta Balea	Vigo	Gastropods	*Patella spp.*
September 1, 2022	Cangas. Punta Balea	Vigo	Echinoderms	*Asterina spp.*
September 1, 2022	Cangas. Punta Balea	Vigo	Crustaceans	*Necora puber*
September 1, 2022	Cangas. Punta Balea	Vigo	Cnidarias	*Anemonia viridis*
September 1, 2022	Cangas. Punta Balea	Vigo	Bivalves	*Mytilus galloprovincialis*
September 9, 2022	Ramallosa. Esteiro Foz	Vigo	Bivalves	*Mytilus galloprovincialis*
September 9, 2022	Ramallosa. Esteiro Foz	Vigo	Bivalves	*Ruditapes decussatus*
September 9, 2022	Ramallosa. Esteiro Foz	Vigo	Bivalves	*Cerastoderma edule*
September 9, 2022	Ramallosa. Esteiro Foz	Vigo	Crustaceans	*Necora puber*
September 9, 2022	Vigo. Praia Canido	Vigo	Echinoderms	*Paracentrotus lividus*
September 9, 2022	Vigo. Praia Canido	Vigo	Gastropods	*Haliotis tuberculata*
September 9, 2022	Vigo. Praia Canido	Vigo	Crustaceans	*Necora puber*
September 9, 2022	Vigo. Praia Canido	Vigo	Crustaceans	*Eriphia verrucosa*
September 9, 2022	Vigo. Praia Canido	Vigo	Gastropods	*Patella spp.*
September 9, 2022	Vigo. Praia Canido	Vigo	Crustaceans	*Palaemon serratus*
September 9, 2022	Vigo. Praia Canido	Vigo	Gastropods	*Monodonta lineata*

n.i. Unidentified

*Coordinates indicated in decimal degrees

Table S2. Multiple Reaction Monitoring (MRM) conditions for lipophilic toxins determination. Precursor ion Q1 = m/z ratio in the first quadrupole, Product ion Q3 = m/z ratio in the third quadrupole, RT (min) = retention time, DP(v) = declustering potential, EP(v) = entrance potential, CE(v) = collision energy, and CXP(v) = collision cell exit potential.

Toxins	Precursor ion Q1	Product ion Q3	RT (min)	DP (v)	EP (v)	CE (v)	CXP (v)	Polarity
**AZA1**	842.3	824.5	8.3	126	10	43	10	positive
**AZA1**	842.3	806.4	8.3	126	10	55	10	positive
**AZA2**	856.3	838.4	8.7	116	10	45	24	positive
**AZA2**	856.3	820.5	8.7	135	10	44	6	positive
**AZA3**	828.3	810.5	8.1	116	10	43	10	positive
**AZA3**	828.3	792.4	8.1	116	10	55	10	positive
**13-desm SPXC**	692.3	674.5	5.2	146	10	45	18	positive
**13-desm SPXC**	692.3	444.3	5.2	146	10	51	14	positive
**OA**	803.4	255.0	7.3	-210	-10	-64	-9	negative
**OA**	803.4	112.7	7.3	-210	-10	-91	-5	negative
**DTX2**	803.4	255.0	7.6	-210	-10	-64	-9	negative
**DTX2**	803.4	112.0	7.6	-210	-10	-91	-5	negative
**DTX1**	817.4	255.0	8.3	-230	-10	-62	-9	negative
**DTX1**	817.4	112.7	8.3	-230	-10	-102	-11	negative
**PTX2**	876.5	823.5	7.7	85	10	35	35	positive
**PTX2**	876.5	213.1	7.7	85	10	52	20	positive
**YTX**	1141.3	1061.4	7.5	-95	-10	-50	-13	negative
**YTX**	1141.3	855.3	7.5	-95	-10	-104	-11	negative

Toxins	Precursor ion Q1	Product ion Q3	RT (min)	DP (v)	EP (v)	CE (v)	CXP (v)	Polarity
**45-OH-YTX**	1157.6	1077.6	6.4	-105	-10	-50	-13	negative
**45-OH-YTX**	1157.6	855.5	6.4	-105	-10	-104	-11	negative
**homoYTX**	1155.6	1075.6	7.5	-95	-10	-50	-13	negative
**homoYTX**	1155.6	869.5	7.5	-95	-10	-104	-11	negative
**45-OH-homo-YTX**	1171.6	1091.6	6.4	-95	-10	-50	-13	negative
**45-OH-homo-YTX**	1171.6	869.5	6.4	-95	-10	-104	-11	negative
**PnTXG**	694.5	676.4	5.7	60	10	40	13	positive
**PnTXG**	694.5	164.1	5.7	60	10	50	13	positive
**GYMA**	508.3	490.4	4.5	60	10	40	13	positive
**GYMA**	508.3	392.4	4.5	60	10	45	13	positive
**13-19diDesMeSPXC**	678.4	660.4	4.8	166	10	41	6	positive
**13-19diDesMeSPXC**	678.4	642.3	4.8	166	10	45	12	positive
**20-methyl SPXG**	706.4	688.4	5.3	166	10	37	6	positive
**20-methyl SPXG**	706.4	670.4	5.3	166	10	47	6	positive

Table S3. LOQs for lipophilic toxins determination with acidic chromatographic conditions.

Toxins	LOQs (μg kg^{-1})
**AZA1**	30

**AZA2**	30
**AZA3**	30
**OA**	30
**DTX1**	30
**DTX2**	30
**PTX2**	30
**YTX***	0.075
**homo-YTX***	0.075
**45-OH-YTX***	0.075
**45-OH-homo-YTX***	0.075
**13-desm SPXC**	30

*Units in mg kg^{-1}
